# Novel Protein‐Rich Bioactive Bioink Stimulates Cellular Proliferation and Response in 3D Bioprinted Volumetric Constructs

**DOI:** 10.1002/adhm.202404470

**Published:** 2025-02-25

**Authors:** Suihong Liu, David Kilian, Anne Bernhardt, Katharina Wirsig, Max von Witzleben, Sarah Duin, Anja Lode, Qingxi Hu, Michael Gelinsky

**Affiliations:** ^1^ Centre for Translational Bone Joint and Soft Tissue Research Faculty of Medicine and University Hospital Carl Gustav Carus Technische Universität Dresden 01307 Dresden Germany; ^2^ Rapid Manufacturing Engineering Center School of Mechatronic Engineering and Automation Shanghai University Shanghai 200444 China; ^3^ Shanghai Key Laboratory of Intelligent Manufacturing and Robotics School of Mechatronic Engineering and Automation Shanghai University Shanghai 200444 China; ^4^ Present address: Engineering Science and Mechanics Department Penn State University University Park PA 16802 USA; ^5^ Present address: Department of Materials Science & Engineering Stanford University Stanford CA 94305 USA; ^6^ National Demonstration Center for Experimental Engineering Training Education Shanghai University Shanghai 200444 China

**Keywords:** bioink, cell response, eggwhite powder, extrusion bioprinting, tissue engineering

## Abstract

3D extrusion bioprinting, a promising and widely adopted technology in the emerging field of biofabrication, has gained considerable attention for its ability to fabricate hierarchically structured, native‐mimicking tissue substitutes with precisely defined cell distributions. Despite notable advancements, the limited availability of suitably bioactive bioinks remains a major challenge, hindering the construction of volumetric tissue substitutes effectively mimicking biological functionality. Therefore, this work proposes a protein‐rich, low‐cost, bioactive bioink: abundantly available eggwhite powder (EWP) is leveraged to functionalize an alginate‐methylcellulose (AlgMC) hydrogel matrix and enhance cellular response. The developed EWP‐supplemented bioinks not only maintain favorable printability and high shape fidelity but also exhibit remarkable bioactivity. Notably, incorporating EWP into AlgMC‐based bioinks enhances shear‐thinning features, thereby improving the viability of encapsulated cells within the bioprinted constructs. The versatility and biofunctionality of EWP in bioprinted constructs are demonstrated using three distinct cell types, encompassing sources such as a stem cell line, human soft skin, and stiff bone tissues. Furthermore, the promising and wide applicability of the EWP‐supplemented bioink for biofabrication is demonstrated exemplarily in core‐shell and multi‐channel bioprinting strategies as a proof‐of‐concept for functional tissue construction. These findings underscore the significant and versatile potential of this novel bioink in biofabrication and biomedical applications.

## Graphical abstract

This work describes a protein‐rich, low‐cost, bioactive bioink, prepared by adding eggwhite powder to functionalize an established alginate‐methylcellulose hydrogel blend and enhance its cellular response. The bioink not only maintains favorable printability and high shape fidelity but also exhibits remarkable bioactivity, thereby improving the viability of encapsulated cells within the bioprinted constructs.

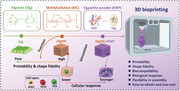

## 1 Introduction

3D bioprinting, a subset of additive manufacturing technologies, has garnered significant attention for its ability to precisely arrange cells, biomaterials, and biologically active factors in a spatially defined manner within 3D constructs for biofabrication of functional tissues.^[^ ^1^, ^2^ ^]^ Extrusion‐based bioprinting, as a more versatile and cost‐effective technology among available methods, enables the layer‐by‐layer deposition of living cells embedded in biomaterials through a nozzle to create complex, scalable, and heterogeneous tissue constructs in defined patterns.^[^ ^3^ ^]^ The viscous building materials used in this process are termed bioinks for cell‐containing materials, or biomaterial inks without cell embedding prior to fabrication.^[^ ^4^ ^]^ Although extrusion bioprinting is widespread due to its excellent compatibility with bioinks of varying viscosities, specific challenges remain, such as the limitations of currently available bioinks for biofabrication of volumetric cell‐laden constructs offering high cell viability while also promoting the adhesion, spreading, proliferation, and lineage‐specific differentiation of cells within bioprinted constructs.^[^ ^5^, ^6^, ^7^ ^]^

The intricate complexity of a native tissue lies in its highly organized cellular architecture and the dynamic interplay between various cell types and the bioactive extracellular matrix (ECM).^[^ ^8^, ^9^ ^]^ Thus, the precise deposition and arrangement of cells, coupled with the engineering of an ECM‐mimicking microenvironment within bioprinted constructs, is imperative for the development of functional tissue substitutes that closely emulate the properties and functions of native tissues. To achieve this, various biopolymer‐based bioinks have been developed in recent years,^[^ ^7^, ^10^ ^]^ protein‐based bioinks such as collagen and gelatin, and polysaccharide‐based bioinks such as alginate (Alg), chitosan, etc. Among them, Alg, a highly cytocompatible biomaterial, exhibits unrivaled potential due to superior cytocompatibility, rheological behavior, and rapid cell‐compatible crosslinking mechanism.^[^ ^11^ ^]^ Since pure Alg cannot form extrudable inks, the addition of methylcellulose (MC), which functions as a thickener to temporarily enhance viscosity during the extrusion process, has been proposed.^[^ ^12^ ^]^ It allows construction of volumetric structures stabilized through calcium ion crosslinking of the Alg network before the un‐crosslinked MC component partly diffuses out leaving behind micropores within fabricated constructs. However, both components exhibit bioinert properties and cannot support the adhesion and spreading of embedded or seeded cells, mainly due to their lack of cell adhesion sites and anionic charge of Alg.^[^ ^11^, ^13^, ^14^ ^]^ These characteristics render AlgMC suitable for applications like cartilage biofabrication,^[^ ^15^ ^]^ where minimal cell interaction is desired, but restrict its use in tissue engineering applications that demand stronger cell‐material interactions for effective cellular integration and tissue functionality. Therefore, developing a versatile and effective strategy to enhance the biological response of the AlgMC‐based hydrogel matrix while maintaining or improving its printability is crucial for its potential application in the biofabrication of functional tissues.

Protein‐based biomaterials exhibit bioactivity and facilitate cellular processes such as attachment, spreading, proliferation, migration, and differentiation, thus making the incorporation of such protein‐rich materials in polysaccharide‐based bioinks a promising strategy to enhance biocompatibility.^[^ ^10^, ^16^ ^]^ Many proteins, however, are dependent on limited resources or require intricate extraction and complicated purification procedures, leading to high costs and undesirable batch‐to‐batch variability. An intriguing and promising alternative is chicken eggwhite (EW, also named albumen), a cheap, ready‐to‐use, readily available, and widely accessible protein‐rich biomaterial, which exhibits promising biological properties and a great potential for tissue engineering applications.^[^ ^17^, ^18^ ^]^ EW is composed of approximately 85% water, 10% proteins, and 5% carbohydrates.^[^ ^17^ ^]^ The proteins, which are the primary functional components of EW, significantly influence its physicochemical and biological properties. These globular proteins are predominantly ovalbumin (54%), followed by ovotransferrin (12%), ovomucoid (11%), ovomucin (3.5%), and lysozyme (3.5%). In recent years, EW has been studied and widely used as a unique supplier of bioactive factors to improve the response of embedded or seeded cells.^[^ ^13^, ^19^ ^]^ For instance, some studies have already shown the feasibility of blending EW in solution with other biopolymers to create constructs with tunable mechanical properties and favorable cell responses.^[^ ^18^, ^20^, ^21^, ^22^, ^23^ ^]^ Additionally, an EW‐based photo‐crosslinkable bioink (optimal EW concentration is 7.5%, w/v) has been synthesized for lithography‐based fabrication.^[^ ^24^ ^]^ Our previous research demonstrated that incorporating EW into alginate‐ or chitosan‐based inks results in 3D printable scaffolds that enhance cell adhesion, proliferation, and vascularization.^[^ ^13^, ^20^ ^]^ Furthermore, to bridge the gap between cell‐free EW‐containing biomaterial inks and cell‐laden EW‐enhanced bioinks for bioprinting, a new EW‐supported bioink (EW protein content around 6%, w/v) was developed, which combines good printability and high shape fidelity with a favorable cell response.^[^ ^19^, ^23^ ^]^ The ability of EW to support positive cell responses is concentration‐dependent, while the protein concentration (around 10%, w/w) in EW solution is constant.^[^ ^17^ ^]^ To the best of our knowledge, the protein content in previously reported biomaterial inks and bioinks derived from EW has not exceeded this baseline level of 10% found in natural liquid EW. Thus, the exploration of bioinks with higher EW protein content and thereby, leveraging protein‐rich eggwhite powder (EWP) as a bioactive supplement to generate nutrient‐enhanced bioinks, boosting cellular response and performance in bioprinted constructs with the aim of overcoming the limitations is promising.

**Figure 1:**
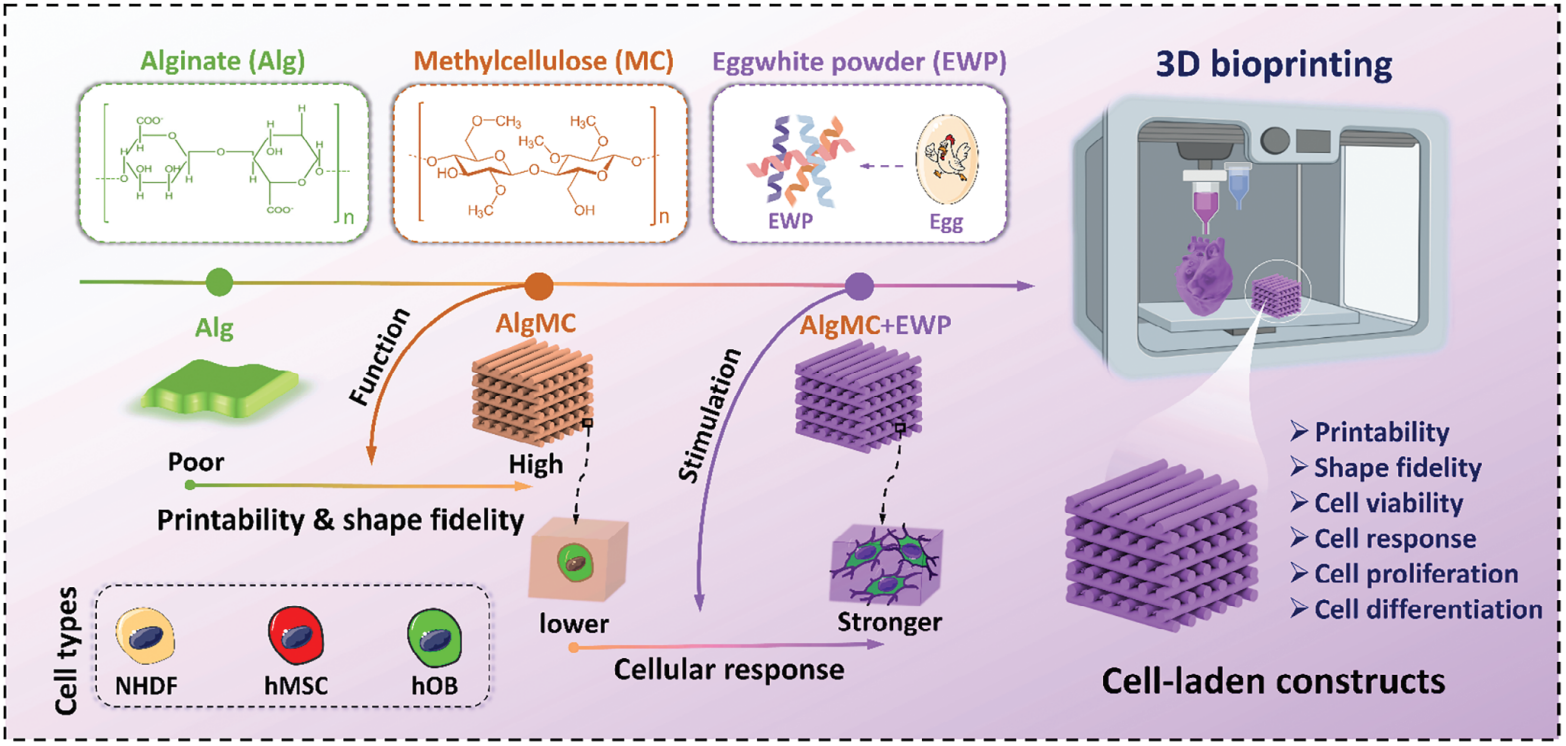
Schematic illustration of the design and preparation strategy of EWP‐supplemented bioinks, three components are combined: the ionically cross‐linkable Alg hydrogel‐forming a stable matrix, the MC whose function is to enable printing with high shape fidelity, and the EWP as a proteinous component to stimulate in cell response, with the objective of augmenting the biological response of an AlgMC‐based hydrogel matrix with favorable printability and shape fidelity and exploring the capability of EWP for stimulation of three distinct cell types (including primary NHDF from soft skin tissue, an immortalized stem cell line: hMSC, and primary hOB from stiff bone tissue) within 3D bioprinted volumetric constructs.

In this study, we propose a novel EWP‐supplemental bioactive bioink, leveraging EWP as a protein‐rich component, with the objective of augmenting the biological response of an AlgMC‐based hydrogel matrix and thereby stimulating cellular behavior (including viability, adhesion, proliferation, and differentiation) within 3D bioprinted constructs. The design and preparation strategy of EWP‐enhanced (bio)inks is illustrated in **Figure** **1**, three components are combined: the ionically crosslinkable Alg hydrogel‐forming a stable matrix, the MC function to enable printing with high shape fidelity, and the EWP as protein component to stimulate cell response. The effect of EWP addition on printability and physiochemical properties of the bioink was investigated. Furthermore, to thoroughly explore the biofunction and stimulatory effect of EWP, the cellular responses of three highly relevant, representative cell types within bioprinted constructs were evaluated: normal human dermal fibroblasts (NHDF) from soft skin tissue, human primary pre‐osteoblasts (hOB) from a patient's femoral head (stiff bone tissue), and an immortalized human mesenchymal stem cell line (hMSC) as a stem cell model. We further investigated the multi‐material multicellular bioprinting flexibility and adaptability of the EWP‐supplemented bioink for complex functional tissue constructs and assessed its potential applications.

## 2 Experimental Section

### 2.1 Materials and Ink Preparation

Alginate (alginic acid sodium salt, Alg, M/G ratio ca. 1:2) from brown algae and methylcellulose (MC, M0512, molecular weight ca. 88 kDa, 4000 cP·s) powders were purchased from Sigma‐Aldrich (Germany) and the dry powders were sterilized by autoclaving at 121 °C for 20 min before use. Organic chicken eggwhite powder (EWP, high gel strength) was kindly provided from OVOBEST Eiprodukte GmbH & Co. KG (Germany). EWP was sterilized by using γ‐irradiation at 25 kGy and stored at 4 °C until use for the preparation of (bio)inks without further treatment.

For the ink preparation, the sterilized EWP was dissolved in phosphate‐buffered saline (PBS, Thermofisher Scientific) in a concentration of 10% and 20% (w/v); and vortexed to obtain homogeneous 10% and 20% EWP‐rich solutions. Afterward, 3% Alg (w/v) and 9% MC (w/v) were dissolved in the prepared EWP‐rich solutions to obtain EWP‐supported AlgMC inks, including AlgMC+10EWP (henceforth referred to AlgMC+EWP in this paper) and AlgMC+20EWP inks. Moreover, 3% Alg and 9% MC (w/v) were dissolved in pure PBS to prepare plain AlgMC ink as an EWP‐free control group.

### 2.2 Source and Expansion of Cells and Bioink Preparation

Normal human dermal fibroblasts (NHDF) were purchased from Promocell (Heidelberg, Germany). Immortalized and undifferentiated human mesenchymal stem cells (hMSC) expressing human telomerase reverse transcriptase (hTERT) were kindly provided by the group of Prof. Matthias Schieker, LMU Munich, Germany.^[^ ^25^ ^]^ NHDF and hMSC were cultured and expanded in DMEM (Dulbecco's Modified Eagle's Medium, DMEM, Biochrom, Germany) with 10% fetal calf serum (FCS, Corning) and 1% penicillin/streptomycin (P/S;, i.e., 100 U mL^−1^ penicillin and 100 µg mL^−1^ streptomycin, Biochrom) in a humidified incubator with 5% CO_2_ at 37 °C. The culture medium was changed twice a week, and cells were passaged at 80% confluency using Trypsin‐EDTA (0.25%).

Harvested NHDF were applied for the respective preparation of AlgMC, AlgMC+EWP, and AlgMC+20EWP bioinks in a density of 3 × 10^6^ cells per gram of ink. Briefly, the harvested 3 × 10^6^ NHDF were suspended in 100 µL DMEM culture medium and added into 1 g of the prepared inks, then gently mixed with a sterilized spatula to obtain NHDF‐laden bioinks immediately prior to bioprinting of cell‐laden scaffolds. In the same way, harvested hMSC were used for the preparation of the respective MSC‐laden AlgMC and AlgMC+EWP bioinks in a density of 5 × 10^6^ cells per gram.

Primary pre‐osteoblasts (hOB) were isolated from the femoral head of a human patient (female, 56 years old) undergoing total hip replacement surgery at the University Hospital Carl Gustav Carus Dresden (Germany) after informed consent (approved by the Ethics Commission of TU Dresden, EK 303082014) as previously described.^[^ ^26^ ^]^ The isolated hOB were cultured and expanded under standard cultivation conditions (37 °C, 5% CO_2_) using α‐MEM (Life Technologies, UK) containing 15% FCS and 1% PS as culture medium until the fourth passage, and used in hOB‐laden AlgMC and AlgMC+EWP bioinks in the same concentration of 3.5 × 10^6^ hOB per gram of prepared inks according to previous work.^[^ ^19^, ^23^ ^]^ The formulation of all bioinks is summarized in **Table** **1**.

**Table 1:** Summary of the formulation of different bioinks.

Cell Types	Bioinks	EWP [%, w/v]	Alg [%, w/v]	MC [%, w/v]	Cell Density [cells/g]
NHDF‐laden bioinks	AlgMC	N/A	3	9	3 × 10^6^
	AlgMC+EWP	10	3	9	3 × 10^6^
	AlgMC+20EWP	20	3	9	3 × 10^6^
hMSC‐laden bioinks	AlgMC	N/A	3	9	5 × 10^6^
	AlgMC+EWP	10	3	9	5 × 10^6^
hOB‐laden bioinks	AlgMC	N/A	3	9	3.5 × 10^6^
	AlgMC+EWP	10	3	9	3.5 × 10^6^

### 2.3 Rheological Measurement

The rheological behavior of cell‐free and NHDF‐laden AlgMC, AlgMC+EWP, and AlgMC+20EWP (bio)inks was analyzed using a rheometer (Rheotest RN 4, Germany) with a plate‐plate setup (diameter: 35 mm) at a distance of 0.1 mm at 25 °C. According to our previous protocol,^[^ ^19^ ^]^ First, the shear‐thinning properties of the prepared cell‐free and NHDF‐laden (bio)inks (Section 2.2) were evaluated by applying a shear rate that progressively increased from 0 to 100 s⁻¹. Subsequently, the shear recovery behavior of the (bio)inks was assessed using a three‐step protocol: an initial low shear rate (5 s⁻¹) within the linear viscoelastic region (LVR) was applied for 120 s, followed by a high shear rate (500 s⁻¹), 100 times greater, exceeding the LVR for 60 s. Finally, the shear rate was reduced back to 5 s⁻¹ within the LVR for another 120 s. The viscosity of the (bio)inks was continuously monitored throughout the experiment. The measurements were repeated three times for each group.

### 2.4 Printability Assessment

The effect of EWP on printability and shape fidelity was investigated for AlgMC+EWP, AlgMC+20EWP, and EWP‐free AlgMC inks. A hollow tube structure (outer diameter: 8 mm, inner diameter: 6.8 mm, height: 10 mm) was designed and printed by utilizing a GeSim printer (BioScaffolder 3.1., Radeberg, Germany) with 22G conical needle (internal diameter: 410 µm), and the corresponding printing parameters are listed in **Table** **2**. The actual height and inner and outer diameter of the formed cylinders were measured, and the shape fidelity of the inks was evaluated by comparing the actual structures with the designed dimensions according to a protocol used in previous studies.^[^ ^27^, ^28^ ^]^

**Table 2:** Summary of the dimension of the structures and corresponding printing parameters.

Structure		Dimension [mm]	Needle [µm]	Ink	Air pressure [kPa]	Printing speed [mm ^−1^s]
Tube structure		Outer diameter: 8 mm Inner diameter: 6.8 mm Height: 10 mm	410	AlgMC	85	9
			410	AlgMC+EWP	70	9
			410	AlgMC+20EWP	45	9
Without support	Nose model	L×W×H = 42×35×18	410	AlgMC+EWP	70	8
	Brain model	L×W×H = 38×28×20	410	AlgMC+EWP	70	8
With support	Heart model	L×W×H = 18×16×32	410	AlgMC+EWP	70	6
			410	MC	180	6
	Hand model	L×W×H = 15×38×43	410	AlgMC+EWP	70	6
			410	MC	180	6

To further evaluate the printability and shape fidelity of the AlgMC+EWP ink, especially for more complex and volumetric geometries, several tissue/organ models with anatomically correct shapes were printed. First, a human nose and a brain model were fabricated without any support structures. Moreover, to further expand our knowledge on the printability and flexibility of the ink, the printing of a human heart and hand models with challenging and over‐hanging features were tested with a sacrificial MC ink (10%, w/v) as support; the MC ink was removed after ionic crosslinking of the AlgMC+EWP structures (10 min in 100 mM CaCl_2_), as previously described.^[^ ^29^, ^30^ ^]^ The dimensions of the structures and corresponding printing parameters are summarized in Table 2.

### 2.5 Mechanical Strength and Morphology Assessment

To investigate the impact of EWP on the mechanical properties of printed scaffolds, EWP‐free AlgMC, AlgMC+EWP, and AlgMC+20EWP scaffolds (cylinder structure with a height of 6 mm and diameter of 10 mm, strand distance 1 mm) were printed as test samples for compression analysis. Post printing, the samples were crosslinked for 10 min in 100 mM CaCl_2_ according to previous work,^[^ ^23^ ^]^ and then incubated in DMEM‐based culture medium under standard cultivation conditions for 1 day to achieve a fully hydrated state. Afterward, the compressive strength of samples was measured using a uniaxial compression approach on a universal testing machine (Zwick‐Roell Z010, Zwick, Germany) equipped with a 100 N load cell at a constant compressive velocity of 0.5 mm min^−1^ at room temperature (RT). The stress‐strain curves were acquired; the corresponding compressive modulus and compressive strength were calculated from the obtained data. Three samples in each group were measured for analysis. In addition, scanning electron microscopy (SEM) images (DSM 982 Gemini, Carl Zeiss AG, Oberkochen, Germany) were captured to evaluate the micromorphology and porosity of printed AlgMC, AlgMC+EWP, and AlgMC+20EWP scaffolds. The printed samples were freeze‐dried and then mounted on stubs and sputter coated with gold before imaging.

### 2.6 Bioprinting of Cell‐Laden Constructs

NHDF‐laden, hMSC‐laden, and hOB‐laden bioinks were prepared separately as described in section 2.2 (Table 1), and transferred into sterilized cartridges with 22G needle (inner diameter: 410 µm) and mounted in the GeSiM printer for biofabrication of cell‐laden constructs. A four‐layered grid structure (L×W×H = 9 × 9 × 1.2 mm) with a layer‐to‐layer orientation of 90° and a strand distance of 2 mm was designed; the corresponding G‐codes were generated by the GeSiM software. Cell‐laden constructs were bioprinted under appropriate printing parameters as shown in **Table** **3**. After bioprinting, the cell‐laden constructs with different cell types (NHDF, hMSC, and hOB) were crosslinked in 100 mM CaCl_2_ for 10 min and incubated in the respective culture media.

**Table 3:** Summary of the printing parameters used for different cell‐laden constructs.

Constructs	Scaffolds	Needle [µm]	Air Pressure [kPa]	Printing Speed [mm ^−1^s]
NHDF‐laden constructs	AlgMC	410	75	7
	AlgMC+EWP	410	70	7
	AlgMC+20EWP	410	40	7
hMSC‐laden constructs	AlgMC	410	60	8
	AlgMC+EWP	410	50	8
hOB‐laden constructs	AlgMC	410	80	6
	AlgMC+EWP	410	55	6

The biofabricated NHDF‐laden AlgMC, AlgMC+EWP, and AlgMC+20EWP constructs were incubated in a humidified incubator with 5% CO_2_ at 37 °C using supplemented DMEM for up to 40 days, and the medium was changed twice a week. At distinct culturing time points, samples were taken for analyses.

The bioprinted hMSC‐laden AlgMC and AlgMC+EWP constructs were cultured under the same conditions as the NHDF‐laden scaffolds for up to 42 days. Moreover, large‐sized hMSC‐laden AlgMC+EWP scaffolds (column structure: diameter of 42 mm and height of 2 mm; cube structure: L×W×H = 16 × 16 × 8 mm) were fabricated and cultured to evaluate the capability for construction of cell‐laden volumetric scaffolds.

The fabricated hOB‐laden AlgMC and AlgMC+EWP constructs were incubated in a basal α‐MEM medium containing 15% FCS and 1% P/S for 14 days under standard cultivation conditions (37 °C, 5% CO_2_). Afterward, osteogenic medium (α‐MEM with 10% FCS, 1% P/S, 10^−7^ M dexamethasone, 10 mM β‐glycerophosphate, and 0.05 mM ascorbic acid 2‐phosphate, denoted as OS^+^ medium) was applied for an additional 14 days until a total of 28 days of culture. The hOB response and activities in scaffolds were evaluated at distinct time points.

### 2.7 Cytocompatibility and Cellular Response in Cell‐Laden Constructs

#### 2.7.1 Cell Viability

The viability of cells embedded in bioprinted constructs was investigated using a Live‐Dead Viability/Cytotoxicity Kit (for mammalian cells, Thermofisher Scientific), whereby the viable and dead cells in the cell‐laden constructs were stained by using calcein AM and ethidium homodimer‐1, respectively, according to the manufacturer's protocol. Briefly, the staining solution was freshly prepared by adding 0.6 µL of calcein AM (live‐cell‐dye) and 1.2 µL of ethidium homodimer‐1 (dead‐cell‐dye) into 1 mL Hanks’ balanced salt solution (HBSS, Thermofisher Scientific, USA). At given time points of cultivation, the cell‐laden constructs were washed in HBSS and incubated in the staining solution for 25 min on a shaker at RT, followed by washing with HBSS three times. The Z‐stack images (thickness: around 400–600 µm) of live and dead cells in the constructs were captured by using a Keyence 9000 fluorescence microscope (Keyence, Japan) and then merged into one maximum‐intensity image. Moreover, quantitative analysis of cell viability in different constructs was performed by using ImageJ software (Fiji 1.44p, National Institutes of Health, USA), and the viability was determined as the ratio of viable cells divided by the total cell number (i.e., the sum of living and dead cells). To further quantitatively analyze the adhesion and spreading of cells in the constructs, the cell area (area covered by viable cells) within constructs was evaluated based on the green channel (calcein‐staining) images of viable cells after 1, 7, and 14 days of cultivation using ImageJ software according to a previously published method.^[^ ^19^ ^]^

#### 2.7.2 Metabolic Activity

To visually observe the cell distribution and metabolic activity of cells in the bioprinted constructs, the cell‐laden constructs were evaluated via a color reaction after incubation in 0.5 mg mL^−1^ 3‐(4, 5‐dimethylthiazol 2‐yl)‐2,5‐diphenyltetrazolium bromide (MTT, Sigma‐Aldrich, USA) in DMEM‐based culture medium under cell culture condition for 3 h. Post incubation, the micrographs of the different cell‐laden constructs were captured using a stereo light microscope (M205C equipped with DFC295 camera, Leica, Germany).

#### 2.7.3 Cell Morphology

To observe the cellular distribution and the effect of EWP on cell morphology in bioprinted constructs, the cell nuclei and cytoskeletons in the constructs were visualized by using DAPI/Phalloidin fluorescence staining according to our previous method.^[^ ^23^ ^]^ Briefly, cells in the constructs were fixed and permeabilized by using 4% paraformaldehyde (VWR Chemicals, France) in HBSS and 0.1% Triton‐X100 (Merck Group) in HBSS. Afterward, the nonspecific binding of antibodies/dyes was blocked by incubation in 1% bovine serum albumin (BSA, Carl Roth, Karlsruhe, Germany) in HBSS. The nuclei and cytoskeletons of cells in the constructs were stained using the DAPI/Phalloidin staining solution, which was prepared by diluting DAPI (0.1 µg mL^−1^, Gibco) and Phalloidin‐iFluor 488 reagent (1 µL mL^−1^, Abcam, USA) reagents into 1% BSA blocking solution under a light‐protected environment. Post‐staining, the z‐stack images (thickness: 400–600 µm) were acquired using a Keyence 9000 fluorescence microscope (Keyence, Japan).

#### 2.7.4 Cell Number and Proliferation

The number of cells in the constructs was measured by quantifying the DNA content according to our previous protocol.^[^ ^19^ ^]^ Briefly, cell‐laden constructs were collected and frozen at −80 °C (*n* = 3 for each condition) until biochemical analysis. Before measurement, the cells were dissociated by dissolving the collected constructs in 100 mM sodium citrate and then lysed by incubation in a water bath at 60 °C overnight, followed by sonication on ice for 10 min. The DNA content was quantified using a Quantifluor dsDNA quantification reagent (Promega, USA) according to the manufacturer's instructions: 10 µL of cell lysates were transferred in duplicate into a black flat 96‐well plate, and then 190 µL of the Quantifluor coloring solution were added in each well. Then, the plate was incubated for 5 min in a light‐protected environment at RT, and fluorescence was read at 485/535 nm (excitation/emission wavelength) with a multifunction microplate reader (Infinite 200 PRO, Tecan, Switzerland).

### 2.8 Assessing the Impact of EWP‐Supported Scaffolds on Osteogenic Differentiation

The osteogenic capability of bioprinted hOB‐laden constructs (as described in section 2.6) was evaluated by gene expression analysis of osteogenic markers. Nine hOB‐laden constructs of each group were collected and frozen at −80 °C separately after cultivation for 28 days, and three scaffolds were pooled for a final replicate number of *n* = 3. To extract RNA, the collected constructs were thawed and dissolved in 100 mM sodium citrate in RNase‐free water at 4 °C for 4 h, and then centrifuged at 3600 rpm at 4 °C for 15 min. Subsequently, the supernatants were discarded and RNA was extracted from the hOB‐containing precipitated pellets using a Qiagen RNeasy Mini Kit (Qiagen, Germany). Next, the extracted RNA was transcribed to cDNA using a high‐capacity cDNA reverse transcription kit (Applied Biosystems, USA) according to the manufacturer's instructions. Afterward, quantitative real‐time polymerase chain reaction (qPCR) was performed using the TaqMan Fast Advanced Master Mix (Applied Biosciences) and TaqMan Gene Expression Assays for bone morphogenetic protein 2 (BMP2, Hs00154192_m1), collagen type I alpha 1 (COL1, Hs00164004_m1), alkaline phosphatase (ALPL, Hs01029144_m1), bone gamma‐carboxyglutamate protein (osteocalcin, BGLAP, Hs01587814_g1), and integrin binding sialoprotein/bone sialoprotein precursor (IBSP, Hs00173720_m1) according to the manufacturer's instructions. The qPCR was run using an Applied Biosystems 7500 fast Real‐Time PCR system. For calculation of the fold changes, β‐actin (ACTB, Hs01060665_g1) was used as a housekeeping gene, comparing the expression of the samples to expression in cells collected at day 0 prior to scaffold fabrication. To analyze the impact of EWP in AlgMC+EWP constructs on osteogenesis, fold changes were normalized to the group of cell‐laden EWP‐free AlgMC constructs cultured under the same conditions.

### 2.9 Expanding the Fabrication Toolbox of EWP‐Supplemented Bioink for the Construction of Complex Functional Tissue Models

To further explore the flexibility and potential application of EWP‐enhanced bioinks, the prepared hMSC‐laden AlgMC+EWP bioinks in Section 2.2 were applied in co‐axial (also named core‐shell) bioprinting and multi‐channel (bio)printing processes for the construction of different functional tissue substitutes. For its application in the core‐shell bioprinting process: hMSC pre‐labeled with DiI (ThermoFisher, Scientific) were used for core bioink (DiI‐labeled hMSC‐laden AlgMC+EWP) preparation, and an AlgMC+EWP bioink laden with non‐labeled hMSC served as the shell; both were coaxially extruded via utilizing a steel‐based co‐axial extrusion module (GeSiM, Germany) to form core‐shell strands and construct specific engineered structures to simulate different tissues. Regarding its application in multichannel (bio)printing processes: a ready‐to‐use calcium phosphate cement (CPC) paste (Innotere, Germany), sterilized by γ‐irradiation (25 kGy), was used as the inorganic phase, and the prepared hMSC‐laden AlgMC+EWP bioink was applied to act as the organic phase; both (bio)inks were printed alternately according to a designed model to construct organic‐inorganic biphasic structures to simulate hard tissues. The printing parameters of core‐shell and multichannel (bio)printing processes are summarized in **Table** **4**.

**Table 4:** Summary of the printing parameters used for bioprinting applications.

Applications	(bio)inks	Outlet diameter [µm]	Air pressure [kPa]	Printing speed [mm ^−1^s]
Core‐shell bioprinting	*Core ink*: DiI‐labeled hMSC‐laden AlgMC+EWP bioink	300	95	3
	*Shell ink*: non‐labeled hMSC‐laden AlgMC+EWP bioink	800	50	
Multichannel (bio)printing	CPC ink	410	210	6
	AlgMC+EWP bioink	410	50	6.5

### 2.10 Statistical Analysis

The results of this study are depicted as mean ± standard deviation (SD). All values were evaluated by single‐factor analysis of variance (ANOVA) with Tukey post‐hoc test while a value of *p *≤ 0.05 was considered statistically significant. Statistical tests were performed using Origin 2017 software (OriginLab, USA). Gene expression data are expressed as fold‐changes (2‐ddCT) ± upper and lower limit. Statistical differences were calculated at the level of dCT values.

## 3 Results and Discussion

### 3.1 Rheological Characterization of EWP‐Enhanced (Bio)Inks

**Figure 2:**
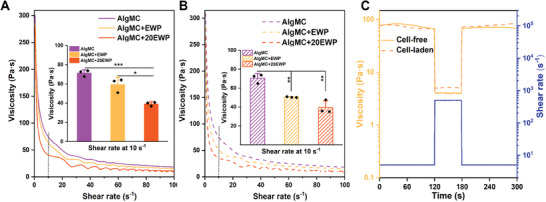
Rheological properties of the prepared (bio)inks. Representative curves of shear thinning behavior of the prepared cell‐free A) and cell‐laden (B; NHDF: 3 × 10^6^ cells per gram of ink) AlgMC, AlgMC+EWP, AlgMC+20EWP (bio)inks during shear ramp experiments (1–100 s^−1^) and the viscosity values of three independent measurements of these (bio)inks with/without NHDF at a shear rate of 10 s^−1^; (insets in A and B) mean ± SD, *n* = 3, *p ≤ 0.05, **p ≤ 0.01, ***p ≤ 0.001). C) Corresponding shear recovery experiments of cell‐free and cell‐laden (NHDF: 3 × 10^6^ cells per gram of inks) AlgMC+EWP (bio)inks during alternating shear rate application of 5 and 500 s^−1^ in defined intervals.

Rheological properties of bioink are the key physicochemical parameters that decisively impact the printability of biomaterials in extrusion‐based bioprinting and can serve as an indicator of their printability and shape fidelity.^[^ ^6^ ^]^ In general, a higher viscosity of shear‐thinning bioink is expected to enhance printability and shape fidelity in support of bath‐free printing, while this typically results in compromised cell viability and motility. Meanwhile, cells, as a fundamental component of bioinks, will occupy a specific volume based on their size and density, potentially influencing the viscoelastic properties of the prepared bioink.^[^ ^31^, ^32^ ^]^ Thus, the rheological properties of cell‐free and cell‐laden (bio)ink compositions (AlgMC, AlgMC+EWP, AlgMC+20EWP) were investigated using shear ramp and shear recovery tests; the results are summarized in **Figure** **2**.

The cell‐free inks (Figure 2A; Figure S1, Supporting Information) and cell‐laden bioinks (Figure 2B) showed promising shear‐thinning behavior, characterized by a decrease in viscosity over a shear rate ramp from 0 to 100 s^−1^. The incorporation of EWP and NHDF (cell density: 3 × 10^6^ cells per gram of ink) did not significantly affect the viscoelastic properties of the (bio)inks, which maintained their adequate shear‐thinning behavior. Interestingly, while the initial viscosity was comparable across all groups (Figure S1, Supporting Information), with increasing shear ramp, the viscosity of EWP‐enhanced (bio)inks was significantly different compared to EWP‐free AlgMC (bio)inks, characterized by a more rapid and substantial decrease in viscosity in response to the applied shear rate ramp, exemplarily analyzed for a shear rate of 10 s^−1^ (Figure 2A,B, inset histograms) for both cell‐free and NHDF‐containing inks (*n* = 3, ^*^ *p* ≤ 0.05, ^**^ *p* ≤ 0.01, ^***^ *p* ≤ 0.001). These results confirm the rheological properties of EW‐supported (bio)inks with a much lower EW concentration (approx. 6%, w/v) observed previously.^[^ ^19^, ^23^ ^]^ Additionally, the shear‐thinning characteristics were progressively amplified with increasing EWP concentration in the (bio)inks (Figure 2A,B), as performed by the rapid decrease in viscosity (Figure S1, Supporting Information).

To clarify, at a certain shear rate, the EWP‐enhanced (bio)inks exhibited lower viscosity, and therefore, a lower shear stress (or printing pressure) is required for stable extrusion, which in turn can contribute to reduced shear stress experienced by the cells within the ink during the bioprinting process and thus improve the viability of cells.^[^ ^33^ ^]^ Notably, a comparison of the viscoelastic properties of cell‐free inks (Figure 2A) with NHDF‐laden bioinks (Figure 2B) indicated that the addition of NHDF at a density of 3 × 10^6^ cells g^−1^ in the bioinks did not show an adverse impact on the shear‐thinning behavior. Furthermore, the shear recovery behavior of AlgMC+EWP with and without NHDF was evaluated during alternating shear rate application of 5 and 500 s^−1^ as shown in Figure 2C. The viscosity in both groups fully recovered after the cycle of low‐high‐low shearing (thixotropic behavior). The investigated density of NHDF in the EWP‐ supplemented bioinks had no adverse impact on viscoelastic properties; similar results were also observed in previously studied MSC‐laden bioinks.^[^ ^23^ ^]^ Overall, after incorporating high concentrations (10‐20%) of EWP into the (bio)inks, they not only maintained promising shear‐thinning and shear recovery behaviors but also showed enhanced responsiveness to shear stimulation; all developed EWP‐containing bioinks showed rheological properties expected to be suitable for extrusion‐based bioprinting of volumetric constructs.

### 3.2 Characterization of Printability and Construction of Complex Volumetric Structures Based on EWP‐Supplemented Inks

**Figure 3:**
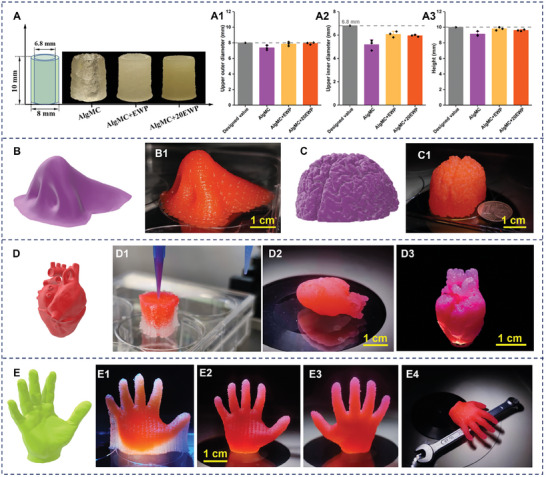
Evaluation of printability and shape fidelity as well as the construction of large‐sized and sophisticated 3D constructs. A) The printability test of AlgMC, AlgMC+EWP, and AlgMC+20EWP inks via printing a designed hollow tube model and the corresponding quantitative analysis of shape fidelity via measuring the resulting top diameter (A1: outer diameter, A2: inner diameter) and height (A3) of printed tube structures, and compared to designed dimensions (mean ± SD, *n* = 3). Fabrication of complex 3D volumetric tissue models (red color added for visualization purposes) B–B1: human nose in realistic dimensions, C–C1: down‐scaled brain model, dimensions listed in Table 2) to further evaluate the good printability of EWP‐supplemented inks by using AlgMC+EWP without any additional support material; and construction of the challenging and sophisticated 3D organ models D–D3: human heart, E‐E4: hand model) by using AlgMC+EWP with dissolved MC (10%, w/v) as supporting ink (white) via multichannel printing process; scale bars represent 1 cm. Design parameters for all geometries are summarized in Table 2.

Based on the results of the rheological characteristics of the developed (bio)inks, their printability and the shape fidelity of printed volumetric structures were evaluated by printing a hollow cylindrical tube.^[^ ^27^, ^28^ ^]^ Representative photographs of the non‐crosslinked cylindrical structures printed with different inks and quantification of their actual dimensions (height; inner and outer diameter at the top of the tube) are presented in **Figure** **3**A. All groups exhibited good printing extrudability and uniformity in extrusion and could form a self‐standing volumetric structure with a hollow core. Notably, the shape fidelity of the printed cylindrical structures with EWP supplementation (AlgMC+EWP and AlgMC+20EWP) was higher compared to the EWP‐free control (AlgMC). Evident diameter decreasing of printed AlgMC tube structure was observed with increasing height (Figure 3A), whereas this phenomenon was mitigated in the AlgMC+EWP and AlgMC+20EWP groups. The incorporation of EWP into the AlgMC matrix could improve the self‐supporting ability of the extruded filaments, thereby enhancing the printability and shape fidelity after EWP addition to (bio)inks (Figure 3A1–A3). The actual outer (Figure 3A1) and inner (Figure 3A2) upper diameters of printed cylindrical structures were closer to the designed values, and also the actual height of the printed structure (Figure 3A3) matched the designed dimension better than that of the AlgMC group, which itself even presents an adequate shape fidelity for clinically relevant dimensions, as determined in many previous studies.^[^ ^12^ ^]^ Overall, the qualitative and quantitative evaluations of printability and shape fidelity demonstrated that incorporating EWP into an AlgMC‐based hydrogel improved the 3D shape fidelity and structural stability of the fabricated constructs.

In addition, 3D printing of anatomically shaped and volumetric structures encompassing challenging geometrical features using the EWP bioink was explored. For this purpose, first, one human nose model with clinically relevant dimensions (Figure 3B,B1) and a down‐scaled, complex human brain model (Figure 3C,C1) were successfully printed with high shape fidelity using our developed AlgMC+EWP ink without any additional support material, exhibiting and maintaining anatomical shape and features. Furthermore, toward more challenging and sophisticated 3D organ models with overhanging features, a complete down‐scaled human heart model (dimensions: L×W×H = 38 × 28 × 20 mm; Figure 3D–D3) and a human hand model (dimensions: L×W×H = 15 × 38 × 43 mm; Figure 3E–E4) were fabricated using AlgMC+EWP with a sacrificial MC support ink using a multichannel printing process. The dimensions and printing parameters of these structures are summarized in Table 2.

Shape fidelity and flexibility of the developed AlgMC+EWP ink was demonstrated in multi‐material multichannel printing with the MC support ink (Figure 3D1,E1) for the construction of complex structures, and the MC support could be easily removed completely as described previously.^[^ ^14^, ^34^ ^]^ The coronary artery features of the printed heart (Figure 3D2,D3), as well as the independent and overhanging finger structures of the hand model (Figure 3E2–E4), were successfully defined and maintained without collapse or breakage. Together, the shape fidelity test and fabrication of complex patterns prove that the novel EWP‐supplemented bioink developed in this work can be utilized with high shape fidelity and self‐supporting ability for 3D (bio)printing of various geometric scaffolds and volumetric constructs in clinically relevant dimensions.

### 3.3 Mechanical and Physical Characterization of 3D Printed EWP‐Rich Structures

**Figure 4:**
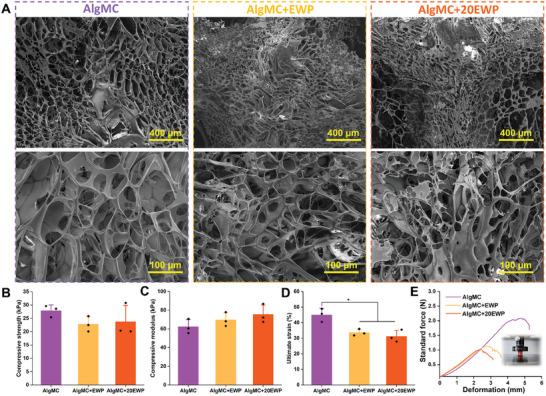
Characterization of micromorphology and mechanical properties of 3D printed structures made of AlgMC, AlgMC+EWP, or AlgMC+20EWP ink: A) Representative SEM micrographs of the microstructure after sample preparation; scale bars represent 400 and 100 µm, respectively. B) Compressive strength and corresponding C) Young's modulus (by compressive testing). D) Ultimate strain at breaking point; and E) the representative force‐deformation curves of different samples, inset: photograph of exemplary sample in compressive testing setup (mean ± SD, *n* = 3, *p ≤ 0.05).

The micromorphology and porosity of constructs printed with the developed hydrogel (bio)inks are critical for cell adhesion, growth, and metabolism.^[^ ^35^ ^]^ Assessing the microporosity of a hydrogel is a challenging task and we assessed their microporous structure after lyophilization by utilizing scanning electron microscopy (SEM) images of the surface of printed AlgMC, AlgMC+EWP, and AlgMC+20EWP structures (**Figure** **4**A). All groups exhibited a highly porous surface and open and interconnected microporosity within the scaffold as reported previously.^[^ ^12^ ^]^ Notably, the addition of EWP (10% and 20%, w/v) did not lead to any adverse effects on the microporous morphology of fabricated structures, and the EW proteins were distributed homogeneously throughout the entire structures.

The mechanical characteristics of the printed structures in an adequately hydrated state like native tissue were evaluated via compression tests after incubation for 1 day in a culture medium under standard cultivation conditions. A representative force‐deformation curve, and the compressive strength and modulus, as well as the ultimate strain until structures broke, are shown in Figure 4B–E. The compressive strength of AlgMC+EWP (22.8 ± 2.9 kPa) and AlgMC+20EWP (23.8 ± 6.1 kPa) were comparable to that of the EWP‐free control, i.e., the AlgMC group (27.9 ± 2.2 kPa), with no significant differences observed between the groups. The corresponding compressive moduli of AlgMC, AlgMC+EWP, and AlgMC+20EWP structures were 62.3 ± 7.7 kPa, 69.8 ± 7.5 kPa, and 75.6 ± 10.0 kPa, respectively. This revealed that the compressive modulus for EWP‐supplemented hydrogel ink increased with the increase in EWP concentration; the value of the maximum strain at breakage decreased (Figure 4D,E). This effect of incorporated EWP might be attributed to the disruption of the crosslinking networks and density of Alg. Notably, the compressive strength and modulus of the EWP‐supplemented inks were further increased compared to the previous AlgMC‐based inks prepared with EW solution.^[^ ^23^ ^]^ While the compressive strength of hydrogel (bio)inks is important for designing robustly engineered scaffolds, their corresponding biological properties are even more crucial for constructing living functional tissue substitutes as the mechanical properties will be complemented and remodeled by secreted extracellular matrix from cells according to specific tissue needs.

### 3.4 The Effect of EWP Addition on Cellular Responses and Viability in 3D Bioprinted NHDF‐Laden Constructs

**Figure 5:**
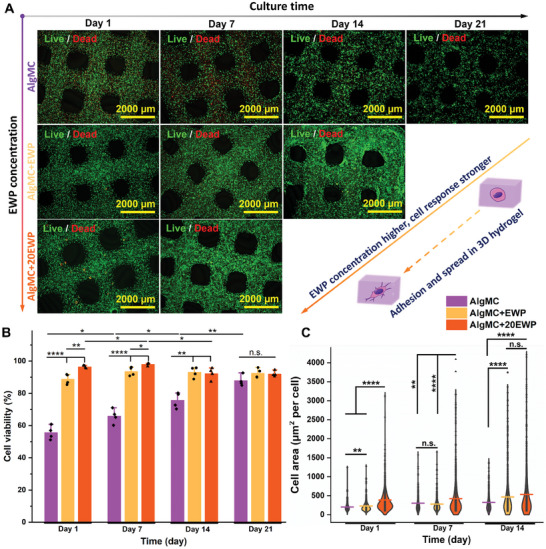
Cellular distribution, viability, and coverage area in bioprinted NHDF‐laden constructs with different EWP concentrations: A) Representative live/dead fluorescence microscope images of embedded NHDF (viable cells in green, dead cells in red) in bioprinted AlgMC, AlgMC+EWP, and AlgMC+20EWP constructs after cultivation for 1, 7, 14, and 21 days to evaluate cell responses with different EWP concentrations, scale bars represent 2000 µm; B) the corresponding cell viability of NHDF in the constructs over 21 days of cultivation (mean ± SD, *n* = 4); and C) the quantification of cell area (coverage area of viable cells) in the constructs using calcein staining images of viable cells after 1, 7, and 14 days of cultivation (*n* = 3, representing the number of images for analysis; a total number of cells in each group of more than 200 cells was applied for statistical analysis; *p ≤ 0.05, **p ≤ 0.01, ****p ≤ 0.0001, n.s. Indicates nonsignificant).

**Figure 6:**
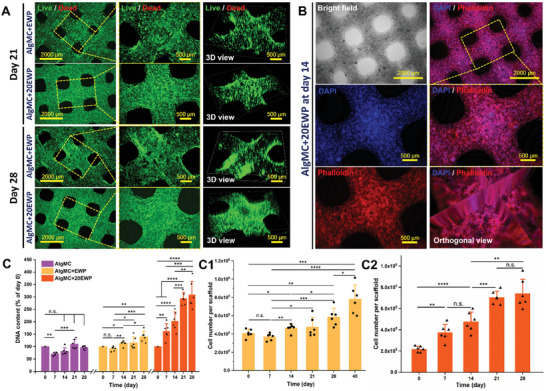
Cellular behavior, morphology, and proliferation of NHDF in bioprinted AlgMC+EWP and AlgMC+20EWP constructs during long‐term in vitro cultivation (28 days). A) Representative live/dead fluorescence microscope images of NHDF in AlgMC+EWP and AlgMC+20EWP constructs after cultivation for 21 and 28 days and the corresponding images with a 3D view to evaluate cellular distribution in the constructs; scale bars represent 2000 and 500 µm. B) Macroscopic view of a bioprinted AlgMC+20EWP construct (brightfield; grayscale) and embedded NHDF in the constructs visualized through fluorescence staining of cell nuclei (DAPI, blue) and actin filaments (phalloidin, red) which were observed after culture for 14 days; scale bars represent 2000 and 500 µm; yellow dashed lines indicate the region of magnification. (C‐C2) Cell number quantification per construct in the AlgMC, AlgMC+EWP, and AlgMC+20EWP groups over the time of culture was determined via quantitative measurements of DNA content: C) DNA content of NHDF in AlgMC, AlgMC+EWP, and AlgMC+20EWP constructs after cultivation for 0, 7, 14, 21, and 28 days; (C1) cell number of NHDF per AlgMC+EWP construct at different time points until 40 days; and (C2) NHDF number in AlgMC+20EWP constructs at different time points of cultivation (mean ± SD, *n* = 3, *p ≤ 0.05, **p ≤ 0.01, ***p ≤ 0.001, ****p ≤ 0.0001, n.s. indicates non‐significant).

To explore the biological functionality and effect of EW proteins on the cell response in the developed EW proteins‐containing constructs, the behavior of NHDF, fibroblasts from human normal skin (as a model cell type from soft tissue), within bioprinted AlgMC+EWP and AlgMC+20EWP constructs were systematically characterized during four weeks of cultivation in terms of cell viability, distribution, adhesion, morphology, and proliferation; bioprinted NHDF‐laden AlgMC constructs served as an EWP‐free control group. All quantitative and qualitative analysis results of these in vitro evaluations are presented in **Figures** **5** and **6** and S2A, and S3 (Supporting Information). The bioprinting parameters of these NHDF‐laden constructs are summarized in Table 3; all the NHDF‐laden constructs with the same dimensions (L×W×H = 9 × 9 × 1.2 mm) were successfully fabricated under such conditions. Notably, the applied air pressure for bioprinting decreased as the EWP concentration in EWP‐containing bioinks had to be increased to maintain a consistent filament size, particularly for the AlgMC+20EWP group (only 40 kPa applied), which can be explained by the rheological properties introduced above (Figure 2; Figure S1, Supporting Information). Similar results were found in our previous study.^[^ ^23^ ^]^ Representative fluorescence micrographs of live/dead‐stained NHDF within bioprinted constructs and a quantitative evaluation of the cell viability are presented in Figure 5 and Figure S2A (Supporting Information). It was evident that the cellular responsiveness of NHDF within the constructs positively correlated with the EWP concentration in the bioink (Figure 5A). In other words, the cellular behavior of NHDF in the bioprinted constructs, including cell viability, adhesion, spreading, and proliferation, was significantly enhanced by EWP addition. Additionally, the bioprinted cell‐laden constructs in all groups maintained their integrity and macroporous structure over the cultivation period, which is crucial for the transport of nutrients, oxygen, and metabolic waste.^[^ ^36^ ^]^

Specifically, on day 1, the cells were homogenously distributed in the entire constructs with a high density in the AlgMC+EWP and AlgMC+20EWP groups, while the viability of NHDF in the AlgMC group was significantly lower than in the other groups (Figure 5A and Figure S2A, Supporting Information). Quantification of viability based on live/dead staining proved this finding (Figure 5B): the viability of NHDF in AlgMC+EWP (88.8% ± 3.2%) and AlgMC+20EWP (96.4% ± 0.8%) was significantly improved compared to those in AlgMC (55.5% ± 4.2%) scaffolds (*n *= 4, ****p ≤ 0.0001); and the viability of NHDF in constructs containing 20% EWP (AlgMC+20EWP) was significantly higher (*n* = 4, **p ≤ 0.01) compared to those in constructs containing 10% EWP (AlgMC+EWP). This might be caused by the contribution of EWP on shear‐thinning behavior as mentioned above (Figure 2; Figure S1, Supporting Information), and cellular protection provided by a protein‐rich environment in EWP‐supplemented constructs. On day 7, the remaining NHDF in AlgMC constructs presented low cell viability (63.3% ± 6.1%) and a more spherical shape while cells in AlgMC+EWP and AlgMC+20EWP groups not only exhibited high cell viability (both > 90%) but also successfully attached and grew in the gel matrix (Figure 5A), evidenced by quantitative evaluation of cell area (coverage area of viable cells staining) within the constructs (Figure 5C). The coverage area per cell within AlgMC+20EWP constructs was significantly higher than in the AlgMC and AlgMC+EWP groups (*n* = 3, **p ≤ 0.01, ****p ≤ 0.0001). Notably, nearly all NHDF in AlgMC+20EWP exhibited spreading and proliferation, whereas this occurred only by day 14 in the AlgMC+EWP constructs (Figure 5A; Figure S2, Supporting Information); this was confirmed by the results of quantitative statistics of cell area (Figure 5C). However, no adhesion, spreading, or growth was observed in the EWP‐free AlgMC group even up to day 21, and cell density within the constructs decreased significantly over time (Figure 5A). This is attributed to the bioinert properties of Alg and MC,^[^ ^11^, ^14^ ^]^ which cannot support the adhesion and spreading of embedded or seeded cells. The significant difference in cellular viability persisted between EWP‐containing groups on day 7 but was lower than on day 1 (*n* = 4, *p ≤ 0.05), and was eliminated by day 14 (Figure 5A). In contrast, the viability of NHDF in AlgMC was significantly lower than in the EWP‐containing groups (*n* = 4, day 7: ***p ≤ 0.0001, day 14: **p ≤ 0.01) and remained so until day 21 (Figure 5B). These findings indicate that the incorporation of EWP can modify and functionalize the AlgMC hydrogel matrix, providing abundant adhesion sites and a conducive microenvironment for cell attachment and spreading. Furthermore, gamma irradiation of EWP does not adversely affect its biological activity, highlighting it as a suitable method for sterilizing EWP‐containing materials.

The cell distribution, density, and morphology of NHDF within EWP‐containing constructs were further investigated over a long‐term cultivation period of more than 4 weeks, and the results are presented in Figure 6A,B. The NHDF‐laden AlgMC+EWP and AlgMC+20EWP constructs maintained their integrity with open macropores after cultivation for 21 and 28 days (Figure 6A), essential for the transport of nutrients, oxygen, and metabolic waste in non‐vascularized 3D systems.^[^ ^37^ ^]^ Moreover, the diameter changes of NHDF‐laden filaments over 21 days, relative to day 1, were analyzed using live/dead fluorescence imaging as an indirect method to further demonstrate the structural stability of bioprinted EWP‐rich constructs with open macroporous architecture (Figure S2B, Supporting Information). Cells within these constructs exhibited high viability, proliferated effectively within the EWP‐containing gel matrix, and nearly achieved complete coverage of the constructs by day 28. Cells were homogenously distributed throughout the constructs, as shown in the 3D view images (Figure 6A). To further explore the morphology and behavior of NHDF within the constructs, the fluorescence microscopic images of fixed constructs with stained cell nuclei (blue) and cytoskeletons (red) after 14 days of cultivation were captured as shown in Figure 6B and Figure S3 (Supporting Information). The nuclei and elongated actin fibers of NHDF within AlgMC+20EWP constructs could be clearly observed (Figure S3, Supporting Information), and interconnected and dense cellular networks were formed due to cell spreading and proliferation in the constructs. Notably, cellular spreading and proliferation in the central regions of the constructs were evident in the orthogonal view images (Figure 6B; Figure S3 (Supporting Information) with high‐resolution images).

Measurement of the DNA content of the NHDF‐laden constructs after 0, 7, 14, 21, and 28 days of cultivation revealed a significant increase in NHDF number in both the AlgMC+EWP and AlgMC+20EWP groups over the cultivation period (Figure 6C). In contrast, the AlgMC group did not exhibit this trend; instead, a reduction in NHDF number was noted (Figure 6C). Furthermore, the cell number of NHDF per AlgMC+EWP construct was measured over long‐term cultivation (up to 40 days) and is presented in Figure 6C1: the NHDF number per construct significantly increased after 14 days of cultivation, and maintained an upward trend through day 40; the NHDF number within the AlgMC+EWP construct at day 40 (i.e., (7.8 ± 1.6) × 10^5^ cells per scaffold) was significantly higher (*n* = 3, *p ≤ 0.001) compared to day 0 (i.e., (4.1 ± 0.5) × 10^5^ cells per scaffold), showing an increase of more than 1.9‐fold. Regarding the AlgMC+20EWP group, NHDF proliferation commenced on day 7 and continued to increase until day 28, with a statistically significant rise in cell numbers observed at each time point (Figure 6C2). The quantity of NHDF within the constructs at day 28 increased to (7.4 ± 1.4) × 10^5^ cells/scaffold, which was significantly higher than the initial count of cells on day 0 (i.e., (2.2 ± 0.3) × 10^5^ cells/scaffold), representing a 3.4‐fold increase. Overall, these results demonstrate that the addition of bioactive EWP was capable of enhancing the biological properties of the AlgMC‐based hydrogel matrix, thereby augmenting NHDF responses in bioprinted EWP‐containing constructs, including improved cell viability, spreading, and proliferation. Based on these results, the AlgMC+EWP (10%, w/v) group was selected for further experiments, to investigate the effect of EWP on cellular responses from other sources.

### 3.5 3D Bioprinting of hMSC‐Laden Constructs and Cellular Dynamics in EWP‐Supplemented Hydrogel

**Figure 7:**
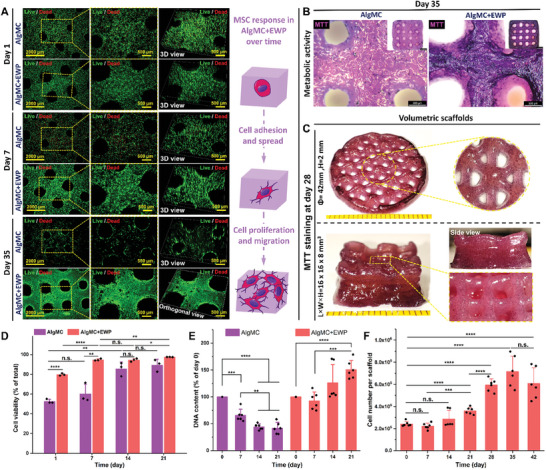
3D bioprinting of hMSC‐laden constructs and characterization of cell responses of embedded hMSC in AlgMC and AlgMC+EWP constructs during long‐term cultivation. A) Representative live/dead fluorescence microscopy images (viable cells in green, dead cells in red) after cultivation for 1, 7, and 35 days and the schematic diagram of cell behavior of hMSC in AlgMC+EWP gels over time of culture; scale bars represent 2000 and 500 µm. B) Representative light microscopic images of MTT‐stained metabolically active hMSC in AlgMC and AlgMC+EWP constructs after culture for 35 days; scale bars represent 2 mm and 500 µm. C) Metabolic activity of hMSC in volumetric AlgMC+EWP constructs after cultivation of 28 days. D) Viability and E) cell proliferation of hMSC in AlgMC and AlgMC+EWP constructs after culture for 0, 7, 14, and 21 days. F) Cell number of hMSC in each of AlgMC+EWP constructs at different time points until culturing for 42 days (6 weeks); (mean ± SD, *n* = 3, *p ≤ 0.05, **p ≤ 0.01, ***p ≤ 0.001, ****p ≤ 0.0001, n.s. indicates non‐significant). Yellow dashed lines indicate the region of magnification.

To further investigate and elucidate the biological functionality of EWP enhancement, the behavior of hMSC within bioprinted AlgMC+EWP constructs, including cell viability, distribution, adhesion, migration, morphology, and proliferation, was systematically characterized during long‐term cultivation, with bioprinted hMSC‐laden AlgMC constructs serving as the control group. All the results of these in vitro observations are presented in **Figures** **7** and S4 (Supporting Information). The bioprinting parameters of these two groups are listed in Table 3, both hMSC‐laden constructs were successfully fabricated under such conditions. Homogenous distribution of hMSC in AlgMC and AlgMC+EWP constructs could be observed in live/dead staining and corresponding 3D view images at day 1 of cultivation (Figure 7A), while the viability of hMSC within AlgMC+EWP constructs (79.8% ± 1.2%) was significantly higher (n = 3, ****p ≤ 0.0001) compared to the EWP‐free AlgMC group (52.7% ± 2.1%) as shown in Figure 7D. Notably, cellular adhesion and spreading within AlgMC+EWP constructs could be prominently observed at day 7, whereas hMSC within AlgMC constructs retained their spherical morphology, with a significant presence of dead cells. Viability of hMSC in both groups increased over time, however, it remained significantly lower in the AlgMC group than in the EWP‐containing group on day 7 (*n* = 3, **p ≤ 0.01). On days 14 and 21, mean hMSC viability remained higher in the AlgMC+EWP group than in the AlgMC group (97.6% ± 0.1% vs 89.3% ± 6.1% at day 21), although not significantly (Figure 7D). After 35 days of cultivation, interestingly, the density and number of hMSC in the AlgMC group markedly decreased, with few live cells remaining, primarily located around the pores and on the surface of the constructs. In contrast, hMSC within AlgMC+EWP constructs maintained good viability, proliferated, and colonized the entire constructs, as shown in live/dead staining images and corresponding orthogonal view images (Figure 7A; Figure S4 (Supporting Information) with high‐resolution images).

The quantity and distribution of metabolically active hMSC in AlgMC and AlgMC+EWP constructs on day 35 were further elucidated through MTT staining (Figure 7B). In addition, two hMSC‐laden volumetric constructs (Φ = 42 mm, H = 2 mm; and L×W×H = 16 ×16 × 8 mm) were bioprinted using the AlgMC+EWP bioink. The hMSC in the EWP‐containing hydrogel matrix maintained high viability and proliferated over 28 days of cultivation, as depicted in the images of MTT‐stained metabolically active hMSC in the constructs (Figure 7C). Subsequently, the proliferation of hMSC in AlgMC and AlgMC+EWP constructs was evaluated by quantifying DNA content, revealing that the number of embedded hMSC in the EWP‐free group significantly decreased over time (Figure 7E), due to the bioinert nature of plain AlgMC, whereas the EWP‐containing group exhibited a substantial increase in hMSC proliferation (Figure 7E), with a significant increase of the cell number in single scaffolds over time (Figure 7F). The number of hMSC within AlgMC+EWP constructs increased to (7.1 ± 1.4) × 10^5^ cells/scaffold by day 35 and was significantly higher (*n *= 3, ****p ≤ 0.0001) compared to day 0 (i.e., (2.4 ± 0.2) × 10^5^ cells/scaffold), representing a 2.95‐fold increase. However, the quantity of hMSC in the constructs on day 42 (i.e., 6 weeks) did not show a further increase, which might be attributed to the space limitation of the constructs for cell growth. Nevertheless, these findings revealed that our novel EWP‐enhanced AlgMC‐based hydrogel system stimulates and augments hMSC behavior within bioprinted constructs as well; this underscores the significant potential of EWP as a bioactive and valuable component in the development of (bio)inks for constructing bio‐functional engineered scaffolds.

### 3.6 Osteogenesis of Bioprinted Primary Pre‐Osteoblasts (hOB) in EWP‐Supplemented Constructs

**Figure 8:**
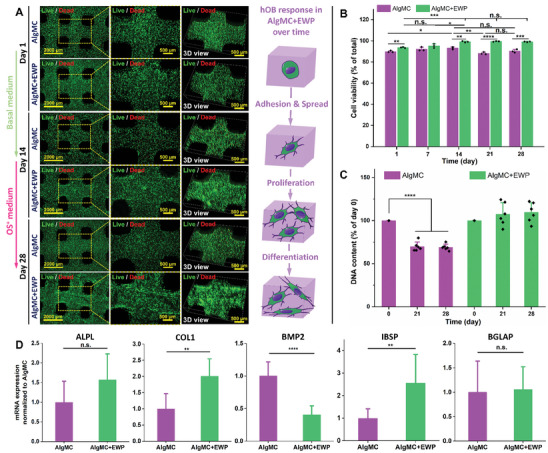
3D bioprinting of primary hOB‐laden constructs and exploration of cellular response and osteogenesis of hOB in AlgMC and AlgMC+EWP constructs. A) Culture schedule and corresponding medium selection for bioprinted constructs, and representative live/dead fluorescence microscopy images of hOB (viable cells in green, dead cells in red) in the constructs after cultivation for 1, 14, and 28 days (scale bars represent 2000 µm and 500 µm, yellow dashed lines indicate the region of magnification), as well as a schematic diagram of cell behavior of hOB in AlgMC+EWP gels over time of culture. B) Corresponding cell viability at days 1, 7, 14, 21, and 28 of cultivation. C) The cell number of hOB in constructs after cultivation for 0, 21, and 28 days was determined via DN A content (mean ± SD, *n* = 3, ****p ≤ 0.0001). D) Osteogenesis on mRNA level of hOB in bioprinted AlgMC and AlgMC+EWP constructs on day 28 (mRNA expression as fold change relative to day 0 and normalized to AlgMC groups, mean ± SD, *n* = 3, **p ≤ 0.01, ****p ≤ 0.0001, n.s. = not significant).

Motivated by the response and behavior of hMSC within the EWP‐containing hydrogel matrix, the effect of such a protein‐rich environment on osteogenic differentiation of primary hOB isolated from a human femoral head of an osteoarthritic patient was further investigated. The hOB‐laden bioprinted constructs were made of AlgMC+EWP bioink and AlgMC as an EWP‐free control group, following the printing parameters listed in Table 3. The results for the viability, morphology, proliferation, and osteogenic differentiation of hOB within both constructs are shown in **Figures** **8** and S5 (Supporting Information). The hOB remained homogenously distributed in bioprinted AlgMC and AlgMC+EWP constructs and maintained high viability after 1 day of cultivation, as shown in the representative live/dead fluorescence microscope and 3D view images (Figure 8A), whereas the viability of hOB within AlgMC+EWP constructs (93.7% ± 0.5%) was significantly higher (*n* = 3, **p ≤ 0.01) than in the AlgMC group (89.9% ± 0.8%) as shown in Figure 8B, confirming results of other cell types NHDF and hMSC described above. The viability of hOB in both groups increased over time. Notably, the viability of hOB within AlgMC+EWP constructs exceeded 99% after 14 days of cultivation, reaching 99.5% ± 0.3% by day 21. This viability was consistently higher than that of the EWP‐free group at the same time points (Figure 8B). Similarly, hOB within AlgMC constructs maintained a roundish morphology throughout the culture period, whereas significant cellular spreading and proliferation were observed in AlgMC+EWP constructs on days 14 and 21 (Figure 8A).

The number of hOB within both types of constructs over time was evaluated by measurement of the DNA content after printing and incubation for 21 and 28 days (Figure 8C; Figure S5, Supporting Information). The number of hOB within AlgMC constructs after 21 and 28 days of cultivation markedly decreased compared to day 0 (*n* = 3, ****p ≤ 0.0001) as shown in Figure 8C. In contrast, the number of hOB within AlgMC+EWP constructs increased consistently over time, although no significant difference was observed. Notably, the number of hOB per scaffold reached (1.26 ± 0.17) × 10^6^ cells/scaffold after 21 days of cultivation (Figure S5, Supporting Information), compared to (1.16 ± 0.09) × 10^6^ cells/scaffold at day 0, indicating an increase of over 1 × 10^5^ cells. Collectively, these results demonstrate that EWP in bioprinted hOB‐laden constructs plays a crucial role in promoting cell adhesion, spreading, and proliferation, indicating the significant potential of EWP in such a hydrogel bioink to enhance the cellular behavior and dynamics of hOB isolated from human bone tissue.

Figure 8D illustrates the osteogenesis of hOB within EWP‐supplemented and EWP‐free AlgMC constructs on mRNA level on day 28 (i.e., day 14 of cultivation in osteogenic medium) relative to the expression in cells collected at day 0. The expression levels of osteogenic markers (ALPL, COL1, BMP2, IBSP, and BGLAP) of hOB cultivated in the EWP‐containing bioink were quantified and analyzed, with the n‐fold changes normalized to the expression when cultivated in plain AlgMC constructs. Notably, the expression of osteogenic markers (COL1 and IBSP) in the EWP‐containing group was significantly upregulated compared to the EWP‐free control (*n* = 3, **p ≤ 0.01), with increases of 2‐fold and 2.6‐fold, respectively. The mean ALPL expression was also observed to be higher than in the AlgMC group but without a significant difference. A significant decrease of BMP2 gene expression in the AlgMC+EWP group (*n* = 3, ****p ≤ 0.0001) could be observed compared to the AlgMC group, whereas no significant differences were detected for the BGLAP marker coding for osteocalcin. Interestingly, the upregulation of IBSP expression of hOB in our previous EW‐containing bioink (EW protein content around 6% (w/v), with the same hOB density of 3.5 × 10^6^ cells/g) was also observed,^[^ ^19^, ^23^ ^]^ along with a consistent trend in ALPL and BGLAP expression in this study. The osteogenic differentiation process involves three key biologically relevant phases: cell proliferation, maturation, and matrix mineralization^[^ ^23^, ^38^ ^]^; the expression of various genes varies significantly across these phases.^[^ ^38^ ^]^ The obtained results on day 28 (with the first 14 days of proliferation in basal medium and the following 14 days of differentiation in culture with osteogenic supplements) indicated that the EWP‐containing bioink allowed osteogenic differentiation of hOB encapsulated in the constructs, and promoted COL1, ALPL, and IBSP gene expression. Our EWP‐supplemented bioactive hydrogel improved the viability of primary hOB in bioprinted constructs and partly supported the osteogenic differentiation of encapsulated cells.

### 3.7 Biofabrication Flexibility of EWP‐Supplemented Bioink for Constructing Complex Functional Tissue Structures

**Figure 9:**
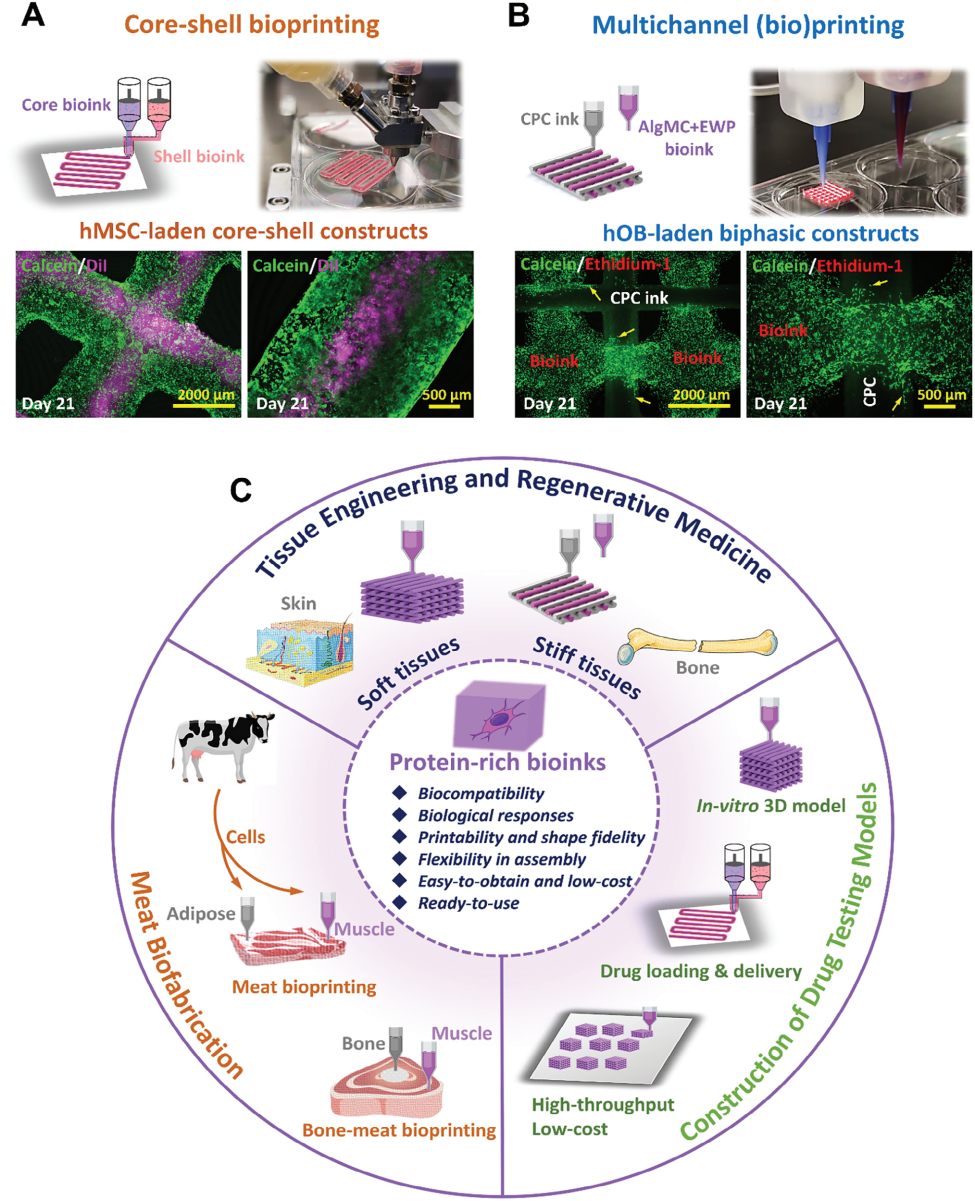
Demonstration of multi‐component printability and flexibility of EWP‐supplemented bioinks to expand the fabrication toolbox toward engineering complex functional tissue constructs. A) Core‐shell bioprinting: as a technical proof‐of‐concept, hMSC as a model cell type was applied, whereby DiI‐labeled hMSC in the AlgMC+EWP bioink served as core bioink, and non‐labeled hMSC in the AlgMC+EWP bioink as shell bioink. The ink exhibited good printability and shape fidelity and it was possible to construct hMSC‐laden core‐shell structures. The MSC maintained high viability and kept their ability to grow in the constructs, as visualized in calcein‐DiI fluorescence images of MSC after cultivation of 21 days. B) Multichannel (bio)printing: as a technical proof‐of‐concept, CPC was applied to serve as inorganic ink, and hOB‐laden AlgMC+EWP bioink as organic bioink to construct hOB‐laden organic‐inorganic biphasic structures; both inks exhibited good printability and good integration between CPC and the AlgMC+EWP bioink was observed. Representative calcein/ethidium‐1 fluorescence microscope images of embedded hOB (calcein: viable cells in green; ethidium‐1: dead cells in red) in biphasic constructs on day 21 of cultivation depict adequate viability, growth, and migration behavior (yellow arrows). Scale bars represent 2000 and 500 µm. C) Outlook of potential applications of the developed easy‐to‐obtain and low‐cost EWP‐supplemented bioinks.

Building on these promising findings and properties of our novel EWP‐supplemented bioactive hydrogel bioink, we further explored its biofabrication boundaries and flexibility for constructing functional cell‐laden tissue constructs for potential bone applications. Two different and equally intriguing biofabrication strategies were applied to further investigate the adaptability and flexibility of the EWP‐supplemented bioink toward the construction of core‐shell constructs and premineralized organic/inorganic constructs, as detailed in our previous studies.^[^ ^23^, ^39^, ^40^ ^]^ The corresponding results are presented in **Figure** **9**A,B, and Figure S6 (Supporting Information): Strategy 1 was the co‐axial (also named core‐shell) bioprinting strategy. As a proof‐of‐concept experiment, hMSC as a model cell type was applied in pre‐labelled form in AlgMC+EWP to simulate the core bioink, and non‐labelled in AlgMC+EWP to serve as shell bioink. Cell‐laden core‐shell filaments and volumetric constructs thereof were successfully formed and fabricated (Figure 9A; Figure S6, Supporting Information) using the EWP‐supplemented bioinks under the printing parameters listed in Table 4, which demonstrated their printability and shape fidelity in core‐shell bioprinting for multi‐cellular assembly. Moreover, the hMSC in biofabricated core‐shell constructs exhibited high viability and cell density as observed in fluorescence images of calcein‐DiI labeled hMSC after cultivation for 21 days (Figure 9A). As strategy 2, multichannel 3D (bio)printing was employed to demonstrate the feasibility of co‐printing the EWP‐containing bioink with an inorganic ink for constructing bone mineral‐like, cell‐laden organic/inorganic biphasic structures. A clinically‐approved and ready‐to‐use calcium phosphate cement (CPC) paste was applied as inorganic ink, and biphasic scaffolds consisting of an hOB‐laden AlgMC+EWP bioink and the CPC were successfully bioprinted (printing parameters are listed in Table 4) as demonstrated in Figure 9B. Meanwhile the live/dead cell staining fluorescence microscopy images of embedded hOB (calcein: viable cells in green; ethidium‐1: dead cells in red) in such biphasic constructs on day 21 of cultivation presented high viability, spreading, and migration behavior (yellow arrows showed in Figure 9B). These results further highlighted the promising flexibility, printability, and integration of the EWP‐supplemented bioink for multi‐cellular multi‐material (bio)printing, via co‐printing with an inorganic CPC ink, by showing that it was possible to construct such cell‐laden and mineralized inorganic/organic biphasic structures to simulate hard bone tissue.

Considering these promising findings together, we envision such high‐protein bioinks and protein‐based biomaterials to play an increasing role in different fields of research. Therefore, we depicted a number of potential applications in tissue engineering, regenerative medicine, and beyond (e.g., food technology for alternative future protein sources) in Figure 9C. EW, a primary protein source for humans, contains a consistent protein concentration of approximately 10% (w/w),^[^ ^17^ ^]^ which was diluted in Hanks’ balanced salt solution and applied for EW‐enhanced bioink preparation in our previous work,^[^ ^19^, ^23^ ^]^ where the final concentration of protein in the bioink is around 6% (w/v), exhibiting promising biological properties for cell viability and spreading, while limited proliferation of cells in bioprinted constructs was exhibited. Exploration of bioinks with higher EW protein is crucial and therefore, EWP, a cheap, ready‐to‐use, easily accessible, and widely available protein‐rich biomaterial, was first introduced in this work and applied to modify and enhance the biofunctionality of an AlgMC‐based biomaterial for bioink development. Results have proved that high protein‐containing bioinks (10% and 20%, w/v) could not only stimulate the cellular response of primary NHDF from soft skin tissue but also augment the response of multipotential hMSC as well as primary hOB from stiff bone tissue. Additionally, its flexibility for constructing cell‐laden mineralized organic/inorganic structures was demonstrated, altogether indicating its great potential in soft and hard tissue repair and regeneration. Moreover, given its good printability, favorable biological response, accessibility, and cost‐effectiveness, we could anticipate it holds significant potential for applications in developing drug testing models, providing useful high‐content platforms to aid drug discovery and personalized therapeutics screening.^[^ ^41^ ^]^ Last but not least, our high protein‐containing bioink, due to its advantages in biofabrication and biocompatibility, could be an ideal candidate for artificial meat production utilizing the biofabrication strategies^[^ ^42^ ^]^ illustrated in Figure 9C. Artificial meat production through 3D bioprinting has emerged as a promising and innovative strategy,^[^ ^42^ ^]^ enabling precise fabrication of cultured meat and attracting significant attention for its potential in sustainable food production. One key challenge in meat biofabrication is the development of suitable bioinks for scalable production.^[^ ^43^ ^]^ Notably, the components of our EWP‐supplemented bioink: Alg, MC, and EWP are well‐established in the food industry, with EWP serving as a common dietary protein source. These attributes position our protein‐rich bioink as a highly promising material for meat biofabrication. The protein‐rich composition of EWP offers dual benefits by supporting both cell growth during bioprinting and maturation and contributing to the nutritional value of the final cultured meat product. This promising application could not only offer a sustainable alternative to traditional meat production but also has the potential to revolutionize the food industry by providing a more environmentally friendly option.^[^ ^43^, ^44^, ^45^ ^]^ While our results suggest promising potential for EWP‐supplemented bioink applications, further studies are necessary to meet specific application requirements, such as optimizing the EWP concentration for particular cell types and further understanding the underlying biological mechanisms as our future goals.

## 4 Conclusion

In this study, we have proposed a protein‐rich bioactive bioink for precise bioprinting of volumetric dimensions with a defined shape, leveraging egg white powder (EWP) as a bioactive protein component, with the objective of augmenting the biological response of cells in an AlgMC‐based hydrogel matrix and thereby stimulating cellular differentiation within 3D bioprinted constructs. The developed EWP‐supplemented bioactive bioinks (AlgMC+EWP and AlgMC+20EWP) exhibited favorable printability, shape fidelity, and cell response compared to an established EWP‐free AlgMC control. The most intriguing findings of our work include the following: 1) Incorporating EWP into an AlgMC‐based hydrogel matrix not only preserves promising rheological behavior but also enhances shear‐thinning responsiveness, thereby mitigating shear stress (or bioprinting pressure) and improves the viability of encapsulated cells within the bioprinted constructs. 2) The cellular response of NHDF within the protein‐rich hydrogel matrix presented a positive correlation with the EWP concentration in the bioink, with cellular activity (viability, adhesion, spreading, and proliferation) increasing as the EWP concentration increased. 3) The EWP‐supplemented bioink demonstrated a broad‐spectrum stimulatory effect on cellular responses, effectively enhancing the activity of three distinct human cell types, including primary NHDF from soft skin tissue, hMSC (an immortalized stem cell line), and primary hOB from stiff bone tissue. 4) Good biofabrication adaptability and flexibility of EWP‐supplemented bioink were demonstrated in core‐shell and multi‐channel bioprinting strategies as the proof‐of‐concept for functional tissue construction. Based on these findings, we further prospected and discussed its potential applications in regenerative medicine and beyond.

## Conflict of Interest

The authors declare no conflict of interest.

## Author Contributions

S.L. performed Conceptualization; Data collection and analysis; Software; Investigation; Methodology; Project administration; Resources; Validation; Visualization; Writing‐original draft; Writing–review and editing; and Funding acquisition. D.K. performed Supervision; Methodology; Investigation; Project administration; Writing–review and editing. A.B. performed Supervision; Formal analysis; Methodology; Writing–review and editing. K.W. performed Methodology; Data collection and analysis; Writing–review and editing. M.W. performed Methodology; Investigation; Writing–review and editing. S.D. performed Investigation; Methodology; Writing–review and editing. A.L. performed Supervision; Formal analysis; Project administration; Methodology; Writing–review and editing. Q.H. performed Supervision and funding acquisition. M.G. performed Supervision; Project administration; Resources; Funding acquisition; Writing–review and editing.

## Supporting information



Supporting Information

## Data Availability

The data that support the findings of this study are available from the corresponding author upon reasonable request.
