# Supplementary material for: Novel Protein‐Rich Bioactive Bioink Stimulates Cellular Proliferation and Response in 3D Bioprinted Volumetric Constructs
Source: Adv Healthc Mater. 2025 Feb 25;14(10):2404470. doi: 10.1002/adhm.202404470 (PMC12004440; doi:10.1002/adhm.202404470)
Supplement: Supplementary file 1 — Supporting Information [file ADHM-14-0-s001.docx]

Supplementary information

**Novel Protein-Rich Bioactive Bioink Stimulates Cellular Proliferation and Response in 3D Bioprinted Volumetric Constructs**

Suihong Liu^1, 2, 3, 5, *^, David Kilian^1, 6^, Anne Bernhardt^1^, Katharina Wirsig^1^, Max von Witzleben^1^, Sarah Duin^1^, Anja Lode^1^, Qingxi Hu^2, 3, 4^, and Michael Gelinsky^1, *^

*^1^Centre for Translational Bone, Joint and Soft Tissue Research, Faculty of Medicine and University Hospital Carl Gustav Carus, Technische Universität Dresden, 01307 Dresden, Germany*

*^2^Rapid Manufacturing Engineering Center, School of Mechatronic Engineering and Automation, Shanghai University, Shanghai 200444, China*

*^3^Shanghai Key Laboratory of Intelligent Manufacturing and Robotics,* *School of Mechatronic Engineering and Automation, Shanghai University, Shanghai 200444, China*

*^4^National Demonstration Center for Experimental Engineering Training Education, Shanghai University, Shanghai 200444, China*

*^5^Current address: Engineering Science and Mechanics Department, Penn State University, University Park, PA, 16802, USA*

*^6^Current address: Department of Materials Science & Engineering, Stanford University, Stanford, CA 94305, USA*

*^*^ Corresponding authors*

*E-mail addresses:*

*Suihong Liu,* [*suihongliu@shu.edu.cn*](mailto:suihongliu@shu.edu.cn)

*Michael Gelinsky,* [*michael.gelinsky@tu-dresden.de*](mailto:michael.gelinsky@tu-dresden.de)


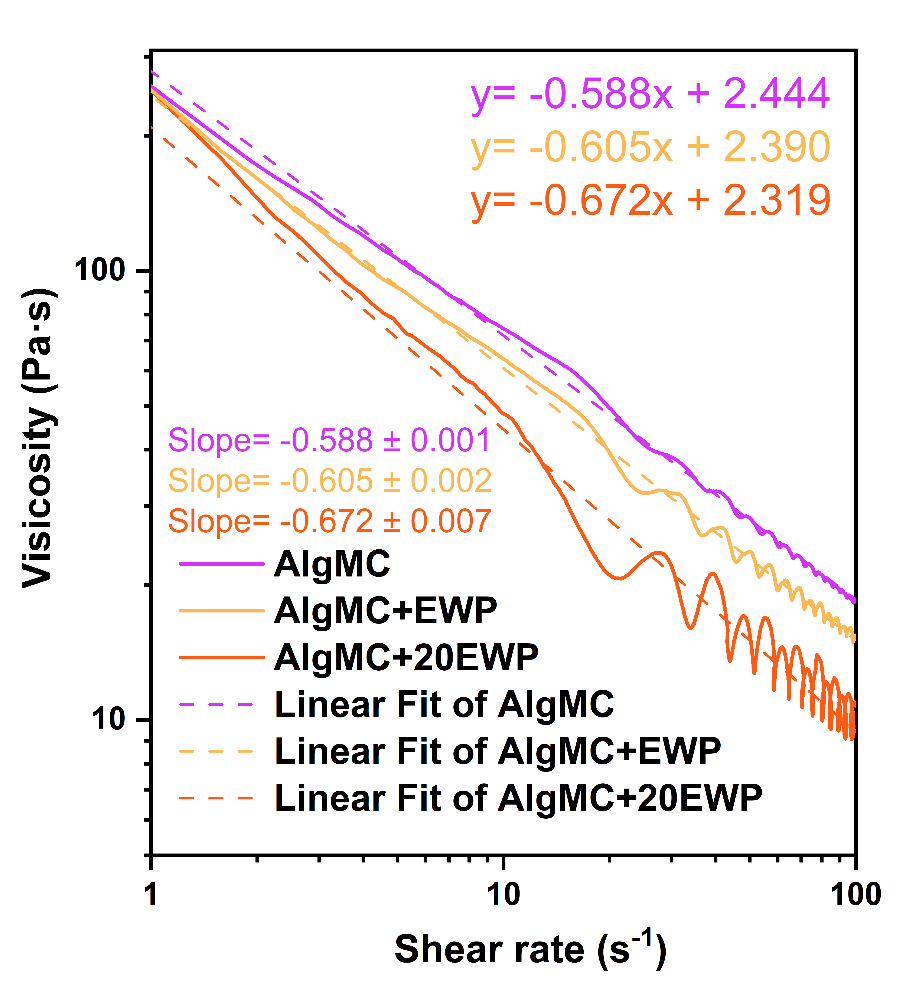


**Figure S1.** Representative curves of the shear thinning behavior of the prepared cell-free inks (AlgMC, AlgMC+EWP, AlgMC+20EWP) during shear ramp experiments (1-100 s^−1^), along with their corresponding linear fit lines, equations, and slope values.


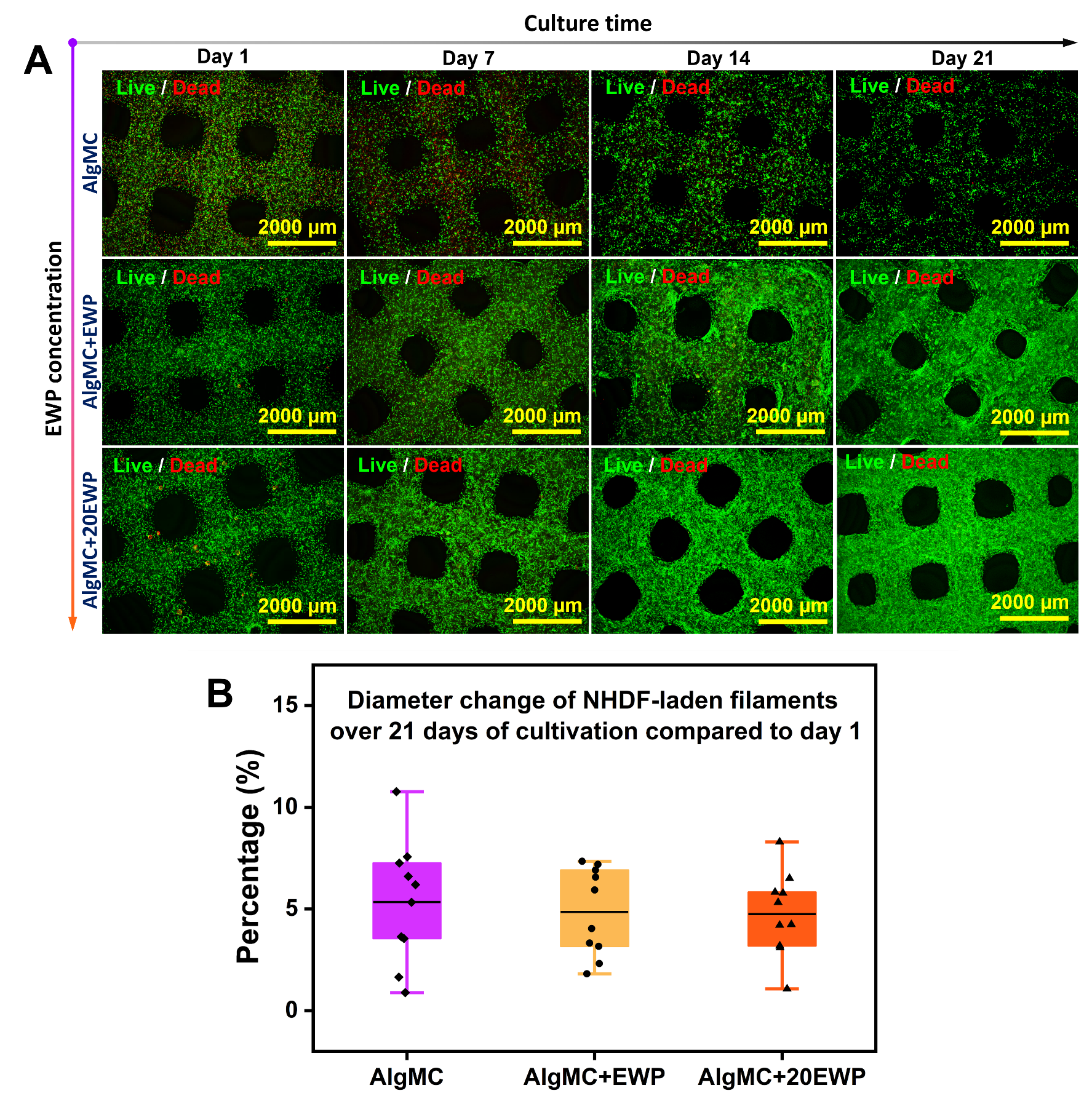


**Figure S2.** (A) Representative live/dead fluorescence microscopy images of embedded NHDF (viable cells in green, dead cells in red) in bioprinted AlgMC, AlgMC+EWP, and AlgMC+20EWP constructs after cultivation for 1, 7, 14, and 21 days to evaluate cell responses with different EWP concentrations; scale bars represent 2000 µm. (B) Diameter changes of NHDF-laden filaments over 21 days, relative to day 1, were analyzed using live/dead fluorescence imaging as an indirect method to assess the effects of ink swelling and degradation on structural stability and macroporous architecture.


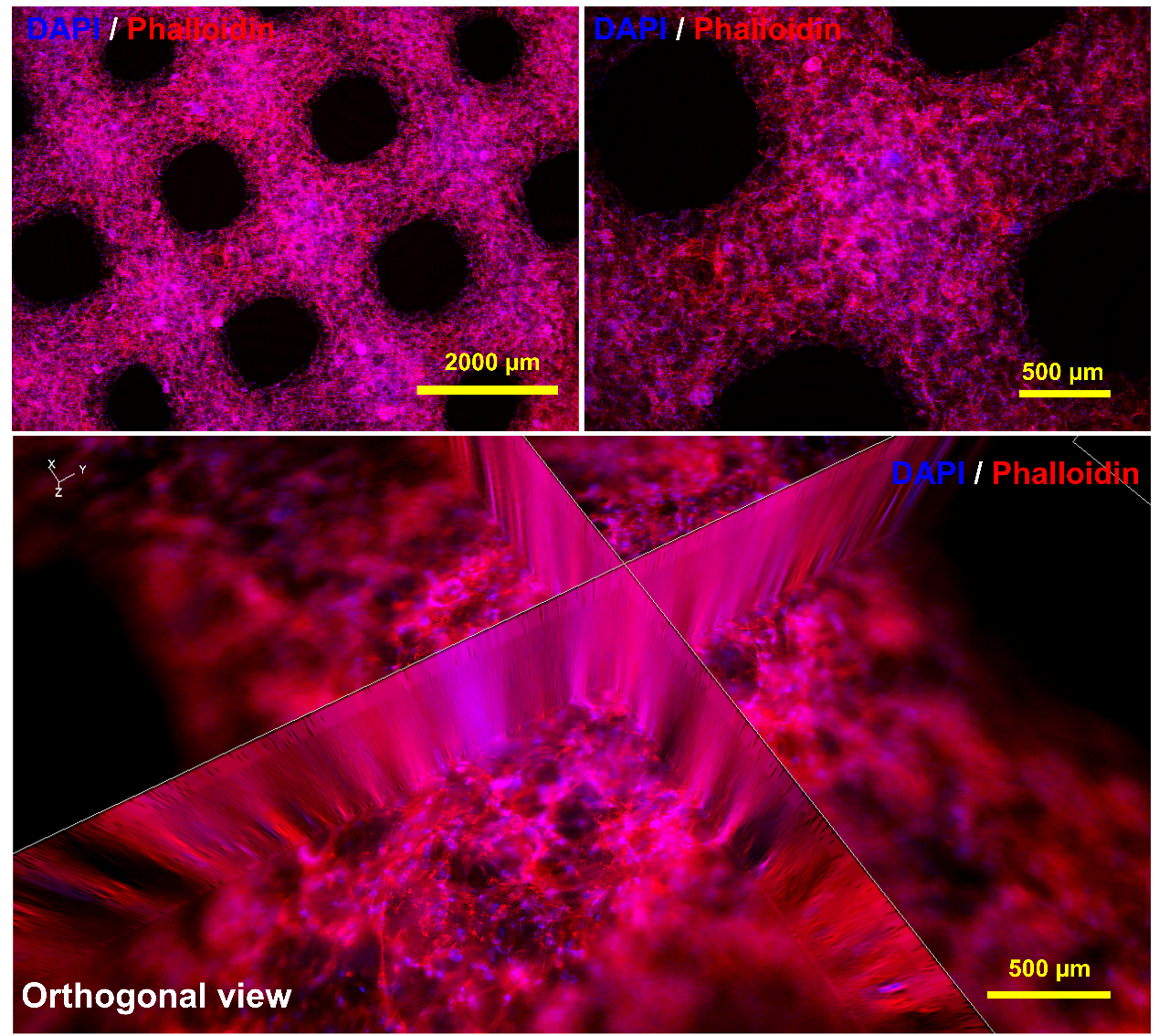


**Figure S3.** High-resolution DAPI (blue: cell nuclei,) and phalloidin (red: cytoskeleton) fluorescence staining images of embedded NHDF in a bioprinted AlgMC+20EWP construct after culturing 14 days to evaluate cellular morphologies; scale bars represent 2000 µm and 500 µm.


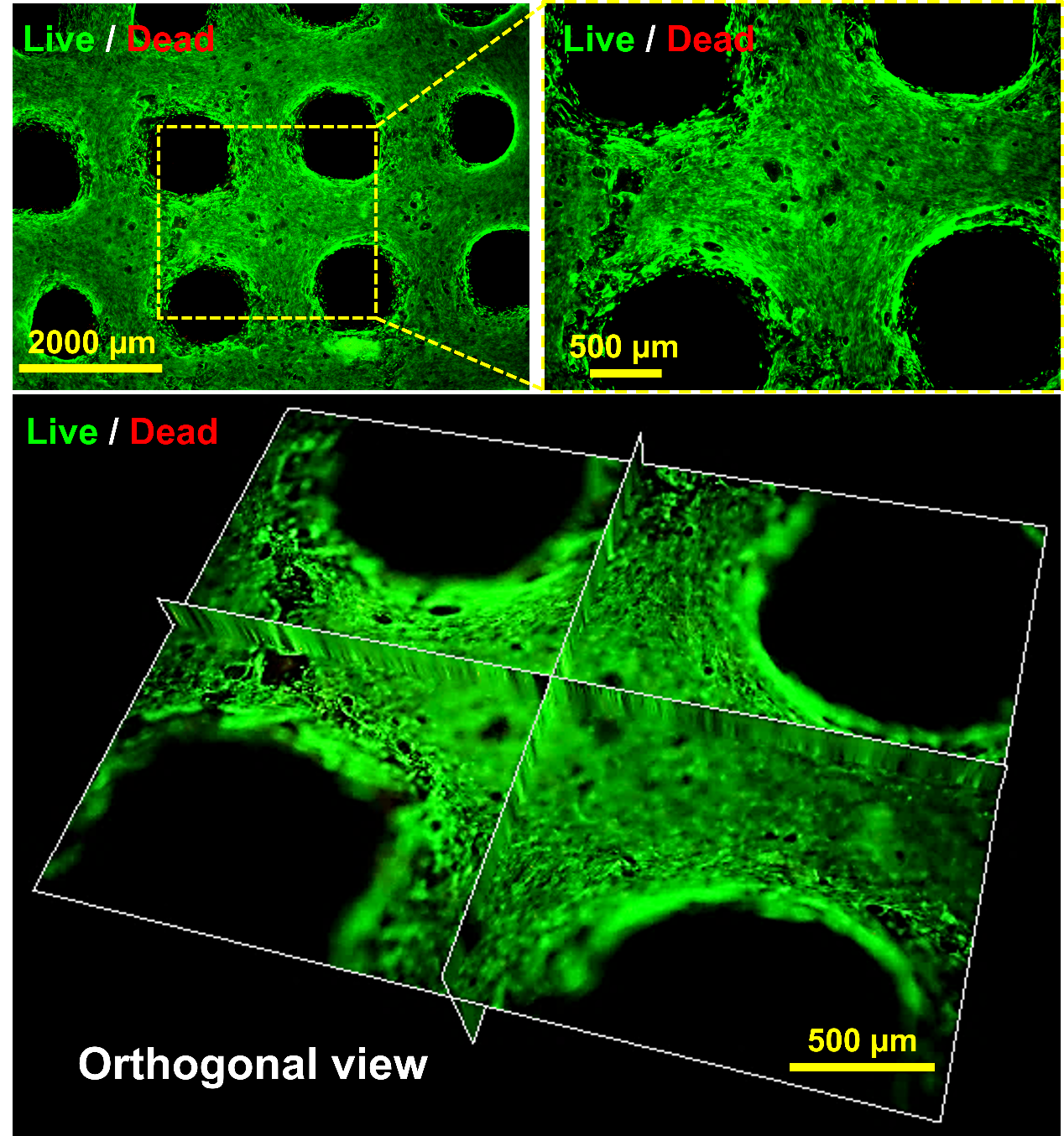


**Figure S4.** High-resolution live/dead fluorescence microscopy and orthogonal view images of embedded MSC (viable cells in green, dead cells in red) in AlgMC+EWP constructs after cultivation for 35 days. Scale bars represent 2000 µm and 500 µm.





**Figure S5.** Cell number of hOB in AlgMC+EWP constructs after cultivation for 0, 21, and 28 days.


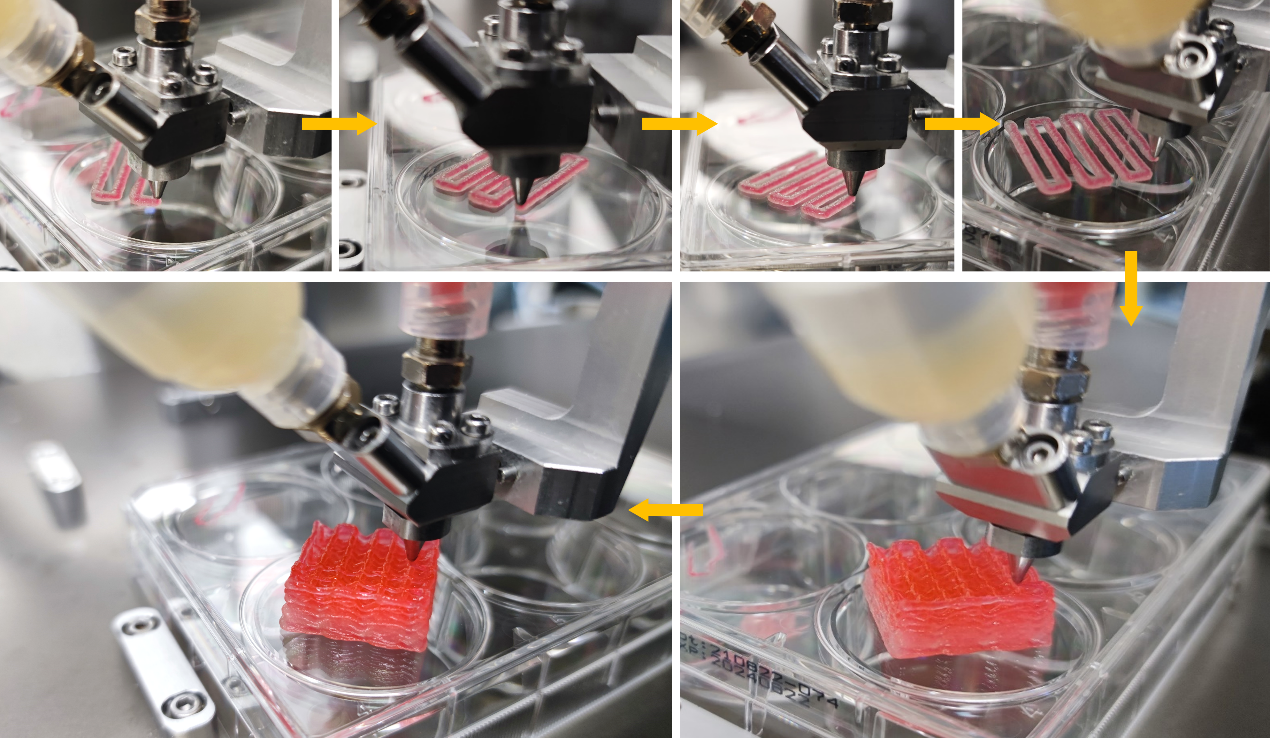


**Figure S6.** Demonstration of printability and flexibility of developed EWP-supplemented bioinks for core-shell (bio)printing: the printing process and fabrication of volumetric constructs with core-shell filaments, where AlgMC + EWP bioink, with or without red dye, served as core and shell ink, respectively.
